# Targeting oncogene-induced senescence in ETV6::RUNX1 pre-leukemic cells

**DOI:** 10.1038/s41420-026-03001-5

**Published:** 2026-03-11

**Authors:** Denise Acunzo, Mayla Bertagna, Giulia Risca, Linda Beneforti, Silvia Bresolin, Stefania Galimberti, Isidro Sánchez-García, Andrea Biondi, Giovanni Cazzaniga, Chiara Palmi

**Affiliations:** 1 Tettamanti Center, Fondazione IRCCS San Gerardo dei Tintori, Monza, Italy; 2 Bicocca Bioinformatics, Biostatistics and Bioimaging Centre (B4), School of Medicine and Surgery, University of Milano-Bicocca, Milan, Italy; 3 Tettamanti Center, Monza, Italy; 4 Pediatric Hematology, Oncology and Stem Cell Transplant Division, Women and Child Health Department, Padua University and Hospital, Padua, Italy; 5 Onco-Hematology, Stem Cell Transplant and Gene Therapy, Istituto di Ricerca Pediatrica Foundation - Città della Speranza, Padua, Italy; 6 Biostatistics and Clinical Epidemiology, Fondazione IRCCS San Gerardo dei Tintori, Monza, Italy; 7 Institute for Biomedical Research of Salamanca (IBSAL), Salamanca, Spain; 8 Experimental Therapeutics and Translational Oncology Program, Instituto de Biología Molecular y Celular del Cáncer, CSIC/Universidad de Salamanca, Salamanca, Spain; 9 School of Medicine and Surgery, University of Milano-Bicocca, Milan, Italy

**Keywords:** Acute lymphocytic leukaemia, Paediatric cancer

## Abstract

The t(12;21)(p13;q22) is the most common chromosomal translocation in pediatric Bcell precursor acute lymphoblastic leukemia (BCP-ALL), occurring in 2-5% of healthy newborns. This alteration generates the *ETV6::RUNX1* (*E::R*) fusion gene, encoding an aberrant transcription factor that is insufficient to directly cause leukemia, but establishes a clinically silent pre-leukemic progenitor not yet fully characterized. We previously showed that E::R expression in the murine pro-B BaF3 cells caused the slowdown of cell cycle progression and increased phospho-histone H2AX levels, both features of Oncogene-Induced Senescence (OIS). This study investigates E::R’s ability to induce senescence in pro-B and immature hematopoietic cells, revealing new therapeutic targets for pre-leukemic cells. We observed that E::R caused a senescence-like phenotype in BaF3 cells, characterized by altered morphology, increased β-galactosidase activity, elevated reactive oxygen species (ROS) and Senescence-Associated Secretory Phenotype (SASP) factor secretion. It dysregulated genes within the p53 pathway, including senescence-related genes, causing the accumulation of p53 protein and alteration in its post-translational modifications. In E::R positive cells, while p53-mediated cell cycle arrest occurred, apoptosis was impaired, providing a survival advantage under genotoxic stress. Multiple therapeutic approaches targeting these vulnerabilities were tested. Senolytics SSK1 and piperlongumine selectively eliminated E::R+ cells by exploiting elevated β-gal activity and ROS levels, respectively. TM5441 leveraged caspase-3 inhibitor PAI-1 upregulation to induce apoptosis. Furthermore, using Sca1-E::R transgenic mice, we validated E::R-induced OIS in the pre-leukemic Lin-Sca1+ immature population and observed reduced pre-B colony formation after SSK1 treatment. These findings demonstrate E::R’s dual role in inducing OIS and conferring apoptosis resistance, highlighting the potential of senescence-targeted therapies to prevent leukemia progression and relapse in E::R carriers.

## Introduction

The t(12;21)(p13;q22) is the most frequent alteration in pediatric B-cells precursor acute lymphoblastic leukemia (BCP-ALL). This chromosomal translocation leads to the generation of the *ETV6::RUNX1* (*E::R*) fusion gene, which encodes for an aberrant transcription factor with a constitutive repressive function [1, 2]. The *E::R* fusion gene arises in utero and is found in 2–5% of healthy newborns [1–4] generating a clinically silent pre-leukemic progenitor characterized by a partial differentiation into B cells, the ability to persist in the organism for many years and increased susceptibility to leukemic transformation [1, 5, 6]. BCP-ALL occurs in about 1% of E::R carriers, following the accumulation of additional mutations [5–7].

The price to pay for the excellent outcome of E::R positive patients is the excessive risk for long-term unwanted comorbidities [8]. In this context, understanding the molecular mechanisms sustaining the pre-leukemic phase and how this converts to frank disease is important not only to intercept leukemia relapses [9], which are often associated with clonal evolution of pre-leukemic clones [10], but also to lay the basis for preventive measures.

We previously showed that the expression of *E::R* oncogene in the murine interleukin-3 dependent pro-B cell line BaF3 slows the cell cycle progression, causing an accumulation of cells in G0/G1 phase and this effect was correlated to an increased expression of cell cycle inhibitors, such as *CDKN1A* (p21^CIP1^) [11]. In agreement with these observations, it has been more recently reported that E::R fusion causes an impairment of the cell cycle in human early B progenitors, particularly in pro-B cells, which appears to resolve in the more mature compartments [12].

The observation that an oncogene in the pre-tumor phase can elicit a growth arrest instead of increasing the cell proliferation rate is not uncommon and this response is often associated with the phenomenon of cellular senescence [13–15].

Cellular senescence was discovered in 1961 when microbiologists Leonard Hayflick and Paul Moorhead described the limited ability to proliferate in culture of normal human fibroblasts [16]. This process is called replicative senescence and is caused by telomere attrition in aged cells. However, premature senescence that does not affect telomeres can also be induced in young cell cultures by various stress stimuli, including the expression of an oncogene, which is called Oncogene-Induced Senescence (OIS).

OIS was initially described in human and murine fibroblasts following the expression of an oncogenic form of RAS [14] and RAF [15]. P53/p21^CIP1^ and p16^INK4A^/Rb pathways are involved in its initiation [17, 18].

Interestingly, the ability of the Runx gene family to cause senescence-like growth arrest in primary murine and human fibroblasts has been described in several reports [19, 20], and in particular RUNX1 and its fusion oncoprotein derivative RUNX1::ETO, that arises as a consequence of the t(8;21) chromosomal translocation in acute myeloid leukemia (AML), are potent inducers of this phenomenon [21].

Cellular senescence was initially proposed as a tumor suppressive mechanism that can block tumor progression at the pre-malignant stage, but nowadays there is emerging evidence that, in certain cellular contexts, cellular senescence can promote cancer development, although the mechanisms are not fully understood [13, 22].

We and other groups described that E::R fusion, in addition to cause the slowdown in the growth of B precursors [11, 12, 23], induces some biomarkers associated with OIS, although not exclusive, such as the accumulation of reactive oxygen species (ROS) [7], a hallmark of senescence-related to mitochondrial dysfunction [24], the increase of DNA double-strand breaks, as revealed by intracellular phospho-histone H2AX flow cytometry assay [7, 23, 24] and the over-expression of the adhesion molecule CD44 and of the chemokine receptor CXCR2, other markers of cellular senescence [23, 25–27].

In this study, we investigated the ability of E::R to induce the OIS state in hematopoietic progenitor cells to identify new therapeutic targets for pre-leukemic cells. To achieve this, we took advantage of two E::R+ pre-leukemic models: the BaF3 Pro B cell-based E::R inducible system [11] and the Sca1-E::R transgenic mouse, in which the expression of E::R is confined to immature hematopoietic Lin-Sca1+ cells [28]. The latter closely resembles the human condition, both in the rare occurrence of BCP-ALL and in its underlying etiology [28].

## Results

### E::R expression induces markers of oncogene-induced senescence in BaF3 pro-B cells

**Fig. 1:**
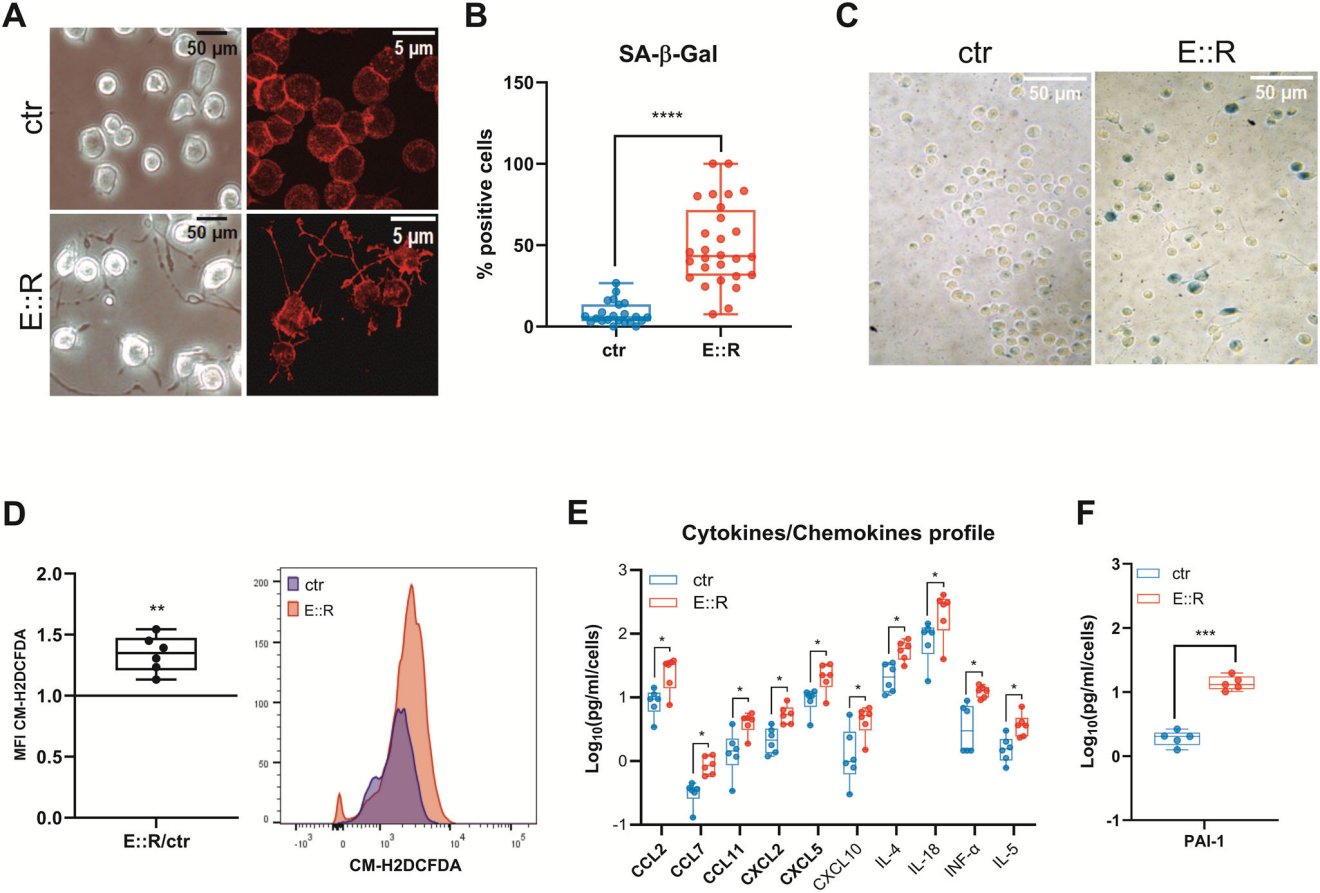
Analysis of markers associated with E::R-induced senescence. **A** Images of E::R+ and control cells were taken with an inverted optical (20× objective and 1.5× digital zoom) (left) or confocal (60× objective and 2.5× digital zoom) (right) microscope. In red the β-actin stained by Alexa Fluor 546 phalloidin. **B** The results represent the percentage of SA β - Gal-positive cells quantified in individual images captured during three independent experiments. Each symbol corresponds to a single image. For each experiment, at least five randomly selected images per well were acquired, and the percentage of positive cells was quantified using ImageJ. Student’s *t* test; ****p < 0.0001. **C** Representative images of E::R+ and control cells taken with an inverted optical microscope at the end of the SA-β-Gal Assay. (10× objective and 1.5× digital zoom). **D** On the left, the boxplot shows the ratio of MFI of the CM-H2DCFDA probe, reflecting intracellular ROS levels, in E::R+ cells compared to control cells of six independent experiments. The MFI of control cells was used as the reference for calculating the fold change. On the right, representative flow cytometry analysis of intracellular ROS in control cells and E::R+ cells. One sample *t*-test; **p < 0.01. **E** The boxplot represents the up-regulated cytokines/chemokines in E::R and control-conditioned media detected from a multiplex assay. In bold are highlighted the molecules that are frequently associated with the SASP profile of senescence cells. The results are represented as the log10 transformation of the normalized concentration. Data analysis was performed using the Wilcoxon-Mann–Whitney test; *q < 0.05. Error bars represent SD. A correction for multiple comparisons with the FDR (5%) approach using the Benjamini, Krieger and Yekutieli method has been performed. **F** Quantification of PAI-1 in E::R and control-conditioned media was performed with ELISA assay. Student’s *t* test; ***p < 0.001.

After induction of E::R expression in the BaF3 model [11], cell morphology changed, with the cells adopting a flattened, enlarged, and vacuolated shape representing the typical morphology of senescent cells [22] (Fig. 1A). Moreover, a higher percentage of SA β-Gal positive cells was observed (% SA β-Gal positive cells: ctr = 8.05 ± 10,61; E::R = 50.02 ± 9.95, *p* < 0.0001, Fig. 1B, C) and, in line with the literature [7, 24], an overproduction of ROS was reported in E::R*+* pro-B cells (fold change of CM-H2DCFDA MFI, E::R *versus* ctr = 1,34 ± 0,15, *p* < 0.01, Fig. 1D). We also investigated the senescence-associated secretory phenotype (SASP), another hallmark of senescent cells [22]. Using a multiplex assay, we analyzed the secretion of 36 cytokines/chemokines (Supplementary Table S1) in the supernatant of cells after induction of E::R expression. We found the up-regulation of 10 different cytokines and chemokines in E::R+-conditioned media, 5 of which, highlighted in the figure, are key components of the SASP [29] (Fig. 1E and Supplementary Fig. S1). In addition, in the E::R+ supernatant, we also detected higher levels of the PAI-1 molecule (Fig. 1F), a key marker and mediator of senescence [30].

These results, combined with our previous observations of replicative restriction, increased DNA double-strand breaks, and over-expression of surface antigens associated with senescence in E::R+ BaF3 cells [11, 23], indicate that E::R expression can promote an OIS response in the pro-B BaF3 cell line.

### E::R alters the expression of p53 target genes involved in the senescence process, causes p53 accumulation and alterations in its post-translational modifications in BaF3 cells

**Fig. 2:**
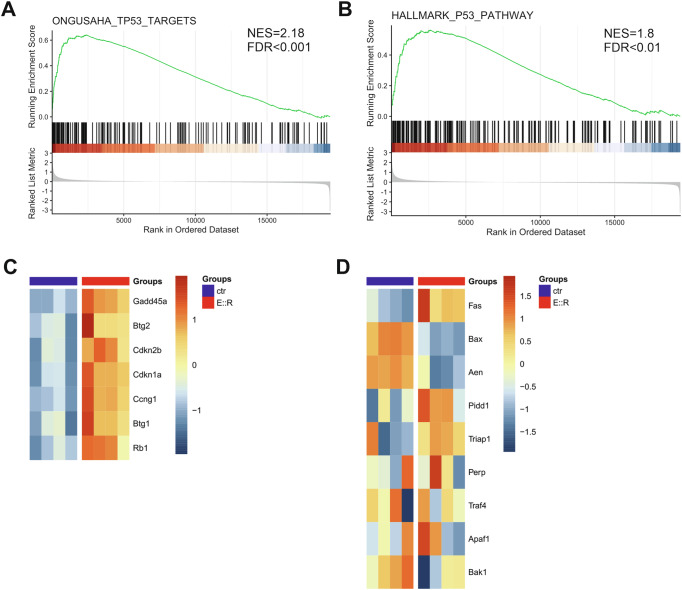
Gene expression analysis of p53 pathway-related genes in BaF3 cells. **A**, **B** GSEA enrichment plots of gene sets associated with the p53 signaling pathway. (**C**) Heatmap of genes from the enriched gene sets in (**A**, **B**), directly involved in cell cycle arrest and significantly upregulated in E::R-expressing BaF3 cells compared to control cells. **D** Heatmap showing the expression of p53-dependent pro-apoptotic genes from the gene sets analyzed in (**A**, **B**), in E::R-expressing BaF3 cells compared to control cells.

The Gene Set Enrichment Analysis (GSEA) applied to the gene expression profile (GEP) of the E::R-inducible BaF3 model [23] identified in E::R+ cells significant enrichments in genes belonging to the p53 pathway, a master regulator of OIS [13] (ONGUSAHA_TP53_TARGETS gene set, FDR q-val = 0 and HALLMARK_P53_PATHWAY gene set, FDR q-val = 0.008, Figs. 2A, B, Supplementary Fig. S2 and Supplementary Tables S2, S3). In particular, we found the upregulation of several genes implicated in cellular senescence. Among these, many are directly involved in the slowdown of the cell cycle, such as *GADD45a, BTG2, CDKN2B, CDKN1a, CCNG1, BTG1* and *RB1* genes [22, 31–34] (Fig. 2C). Notably, *GADD45A* also plays a role in the regulation of the p38 MAP kinase pathway, which is a key mediator of stress-induced senescence [32, 35]. In contrast, aside from the upregulation of *FAS*, we did not observe increased expression of p53-dependent pro-apoptotic genes, despite their presence in the analyzed gene sets, such as *PIDD1*, *TRIAP1*, *PERP*, *TRAF4*, *APAF1*, *BAK1, BAX*, and *AEN* [36, 37] (Fig. 2D).

**Fig. 3:**
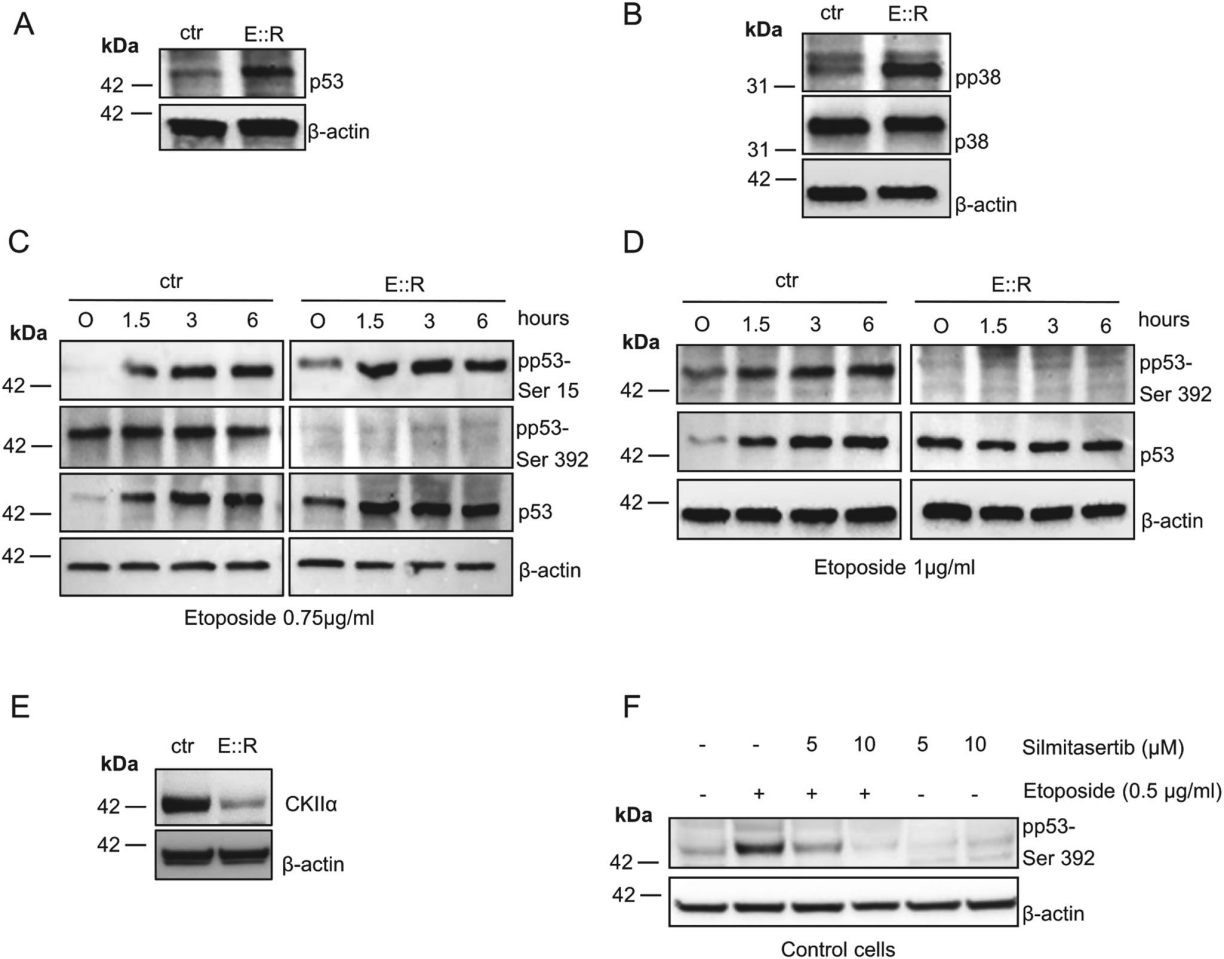
Analysis of p53 and p38 MAPK pathways in E::R+ and control cells. **A** Western blot analysis of total p53 protein in basal condition in E::R+ and control cells. **B** Western blot analysis of total and phosphorylated form (pp38-Thr180/Tyr182) of p38 MAPK protein in E::R+ and control cells. **C** Western blot analysis of pp53-ser15 and pp53-ser392, in untreated condition and after different time points of etoposide treatment at 0.75 µg/ml in E::R+ and control cells. **D** Western blot analysis of pp53-ser392 in untreated condition and after different time points of etoposide treatment at 1 µg/ml in E::R+ and control cells. **E** Western blot analysis of CKIIα protein in control and E::R+ cells in untreated condition. **F** Western blot analysis of pp53-serine 392 in control cells in untreated condition and after 2 days of etoposide and/or silmitasertib treatment.

We then investigated p53 protein levels and its modifications. The accumulation of the p53 protein in E::R*+* cells compared to control cells (Fig. 3A and original image 3A) goes along with the increased phosphorylation levels of the active form of p38 MAPK (pp38-Thr180/Tyr182), while the total level of p38 remains unchanged (Fig. 3B and original image 3B), a condition which promotes the senescence response [21, 38, 39].

Next, we focused on two phosphorylated forms of p53: the pp53-S15 and the pp53-S392 (corresponding to murine Ser-389), both of which play key roles in maintaining p53 stability and triggering proper DNA damage and apoptotic cascade response after stresses [40, 41].

We didn’t detect any defects in the ability of E::R cells to phosphorylate p53 at the S15 site, both under basal conditions and following treatment with etoposide, a DNA topoisomerase II inhibitor that induces DNA damage [42], for 1.5, 3 and 6 h. On the other hand, we observed that control cells exhibit phosphorylation on serine 392 under basal conditions, which further increases after etoposide treatment. In contrast, E::R+ pre-leukemic cells showed no detectable pp53-S392 form under any of the conditions examined (Fig. 3C and original image 3C). Notably, even at higher etoposide concentration (1 μg/ml), phosphorylation at 392 serine remained absent in E::R cells, while an increase in this form was clearly observed in control cells after treatment (Fig. 3D and original image 3D).

Interestingly, the defect in serine 392 phosphorylation of p53 in E::R*+* cells correlates with a lower level of the CKIIα protein (Fig. 3E and original image 3E), a serine/threonine kinase known to target this specific residue in some cellular contexts [43]. This is further sustained by the reduction in the activation of serine 392 phosphorylation by etoposide when control cells were treated for 48 h with silmitasertib (CX4945), an inhibitor of CKIIα activity (Fig. 3F and original image 3F). Furthermore, in E::R+ cells, we found the up-regulation of the gene *LMNA* that encodes Lamin A, the major component of the nuclear lamina which has been reported to attenuate the activity of CKIIα kinase and promote the senescent response [44] (log2 fold change of *LMNA* mRNA, E::R *versus* ctr = 1.2 ± 0.02, FDR < 0.05, Supplementary Fig. S3).

These results suggest that E::R+ pre-leukemic cells display an impaired p53 functionality, especially in the apoptotic signaling pathway mediated by the serine 392 phosphorylation, and the reduced protein level and activity of the CKIIα kinase may contribute to this dysfunction.

### E::R expression caused a growth advantage in the presence of DNA damage-inducing stimuli

Based on the observation that, even in the presence of DNA damage-inducing stimuli, E::R+ cells don’t exhibit phosphorylation at serine 392 of p53, we performed a competitive assay by co-culturing control and E::R+ cells in the presence of DNA injury triggers to evaluate whether the pre-leukemic cells gain a survival advantage under genotoxic stress compared to the control cells.

**Fig. 4:**
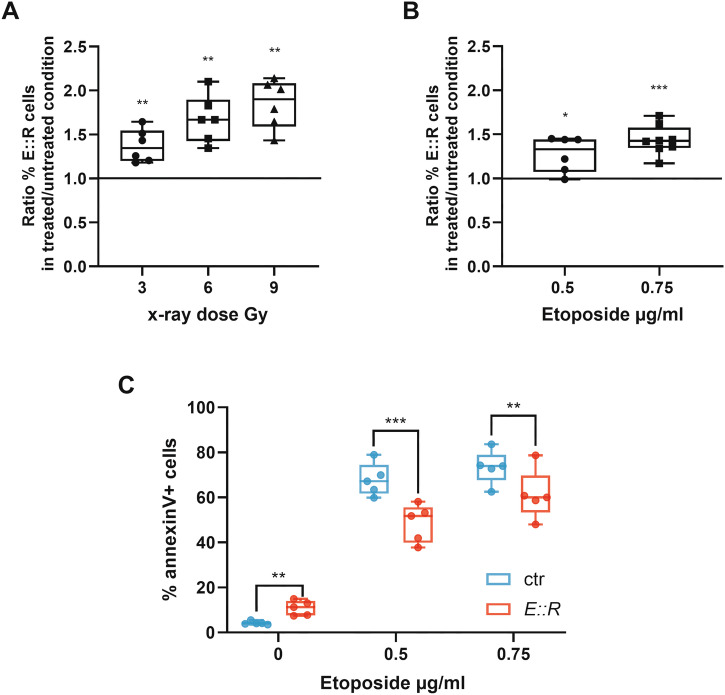
Analysis of competitive growth and apoptotic response in presence of genotoxic stimuli. Competitive assay performed by co-culturing together E::R+ and control cells in untreated condition, after treatment with etoposide (**A**) or after x-ray irradiation (**B**). Boxplots represent the mean of the ratio between the percentage of E::R+ cells treated with eto or x-ray and the percentage of E::R+ cells in the untreated condition of six independent experiments. The percentage of E::R+ cells in the untreated condition was considered as reference and indicated with a line in the graph. One sample *t* test, a correction for multiple comparisons with the false discovery rate (FDR = 5%) approach using the Benjamini, Krieger and Yekutieli method has been performed *q < 0.05, **q < 0.01, ***q < 0.001. **C** Boxplots represent the percentage of annexin V+ cells after 2 days of treatment with different concentrations of etoposide of E::R+ and control based on four independent experiments. Student’s paired *t*-test; a correction for multiple comparisons with the false discovery rate (FDR = 5%) approach using the Benjamini, Krieger and Yekutieli method has been performed; **q < 0.01, ***q < 0.001.

After 2 days of treatment with different doses of etoposide or x-ray, we observed a statistically significant increase in the percentage ratio of treated E::R+ cells compared to the untreated condition (irradiation: 3 Gy = 1.37 ± 0.18, *q* < 0.01; 6 Gy = 1.68 ± 0.26, *q* < 0.01; 9 Gy = 1.85 ± 0.27, *q* < 0.01; etoposide: 0.5 μg/ml = 1.27 ± 0.2, *q* = 0.02; 0.75 μg/ml = 1.44 ± 1.17, *q* < 0.001, Figs. 4A, B).

Following etoposide treatment, we detected a lower mortality of E::R cells compared to control cells (% annexin V+ ctr *versus* E::R cells: 0.5 μg/ml etoposide = 68 ± 7.2 *versus* 48 ± 8.4, *q* < 0.001; 0.75 μg/ml etoposide = 73 ± 7.5 *versus* 61 ± 11, *q* < 0.01, Fig. 4C); thus confirming that the growth advantage of the E::R+ cells was due to a higher resistance to the genotoxic stress-induced apoptosis.

### SSK1, TM5441 and piperlongumine treatment specifically targets E::R+ pre-leukemic senescent cells

**Fig. 5:**
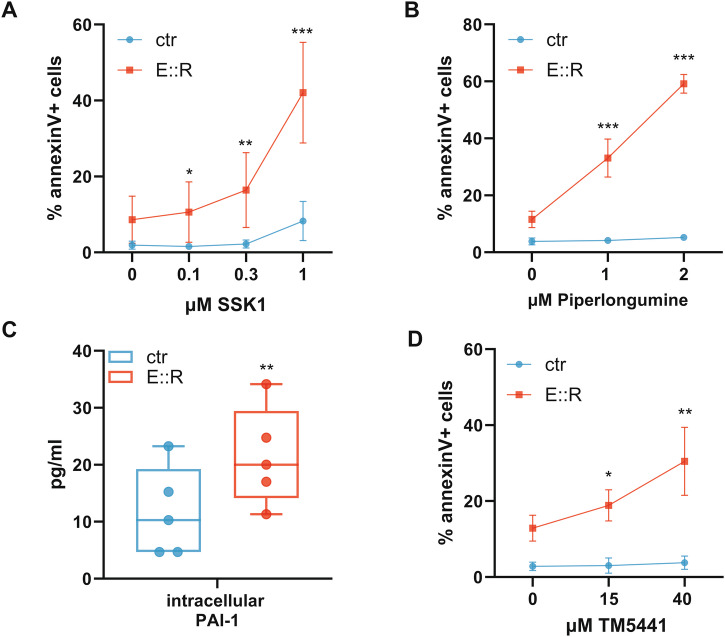
Selective targeting of E::R expressing BaF3 cells using senolytic strategies. **A** Annexin V assay of E::R and control cells after 2 days of treatment with SSK1. The curve represents the mean (±SD) percentage of annexin V + E::R cells and annexin V+ control cells after the treatment with different concentrations of SSK1 in six independent experiments. Student’s paired *t*-test was performed vs their own untreated, a correction for multiple comparisons with the false discovery rate (FDR = 5%) approach using the Benjamini, Krieger and Yekutieli method was applied; *q < 0.05; **q < 0.01; ***q < 0.001. **B** Annexin V assay of E::R and control cells after 2 days of treatment with PL. The curve represents the mean (±SD) percentage of annexin V + E::R cells and annexin V+ control cells after the treatment with different concentrations of PL in five independent experiments. Student’s paired *t*-test was performed vs their own untreated, a correction for multiple comparisons with the false discovery rate (FDR = 5%) approach using the Benjamini, Krieger and Yekutieli method has been performed; ***q < 0.001. **C** Quantification of PAI-1 in lysates of E::R+ and control cells was performed with ELISA assay. Student’s paired *t* test, **p < 0.01. **D** The curve represents the mean (±SD) percentage of annexin V + E::R cells and annexin V+ control cells after the treatment with different concentrations of TM5441 in six independent experiments. Student’s paired *t*-test was performed vs their own untreated, a correction for multiple comparisons with the false discovery rate (FDR = 5%) approach using the Benjamini, Krieger and Yekutieli method was applied; *q < 0.05; **q < 0.01.

To eradicate the pre-leukemic cells, we considered exploiting their observed senescent state. First, leveraging the high activity of SA β-Gal in E::R+ cells, we treated them with SSK1, a senolytic prodrug specifically cleaved by this enzyme into cytotoxic gemcitabine and thus able to induce apoptosis only in senescent cells [45]. After two days of treatment, SSK1 induced significant cell death in E::R-expressing cells (% annexin V+ E::R cells: 0.1 µM SSK1 = 10.6 ± 7.9, *q* = 0.01; 0.3 µM SSK1 = 16.4 ± 9.4, *q* < 0.01; 1 µM SSK1 = 42 ± 13.2, *q* < 0.001), while control cells were slightly affected only by the higher dose (Fig. 5A).

Next, we exploited the elevated levels of ROS observed in E::R+ cells (Fig. 1D) by investigating the effects of piperlongumine (PL), a natural senolytic agent known to enhance the sensitivity of senescent cells to oxidative stress, leading to a further increase in ROS levels that ultimately pushes these cells toward apoptosis [46]. Following two days of PL treatment, we showed that this agent effectively eliminated the pre-leukemic cells, without impacting control cells (% annexin V+ E::R cells: 1 µM PL = 33 ± 6.7, q < 0.001; 2 µM PL = 59 ± 3.3, *q* < 0.001, Fig. 5B).

Additionally, we investigated the targeting of the SASP factor PAI-1, a mediator of senescence that also exerts an important intracellular antiapoptotic function by preventing the activation of caspase-3 [47]. Notably, PAI-1 was found up-regulated in E::R+ cells not only in its secreted form (Fig. 1F), but also at the intracellular protein level (ctr = 11.6 ± 7.8 pg/ml; E::R = 24.1 ± 8.6 pg/ml, *p* < 0.01, Fig. 5C). After two days of treatment with the PAI-1 inhibitor TM5441, we observed that this molecule selectively and effectively induced cell death in the pre-leukemic cells (% annexin V+ E::R cells: 15 µM TM5441 = 18.8 ± 4, *q* = 0.02; 40 µM TM5441 = 30.5 ± 8.9, *q* < 0.01, Fig. 5D).

### E::R expression causes OIS phenotype in Lin- Sca1+ immature progenitors

We evaluated the ability of E::R to induce OIS and the potential to exploit this characteristic for eradicating pre-leukemic cells in a recently developed Sca1-E::R transgenic mouse model [28].

**Fig. 6:**
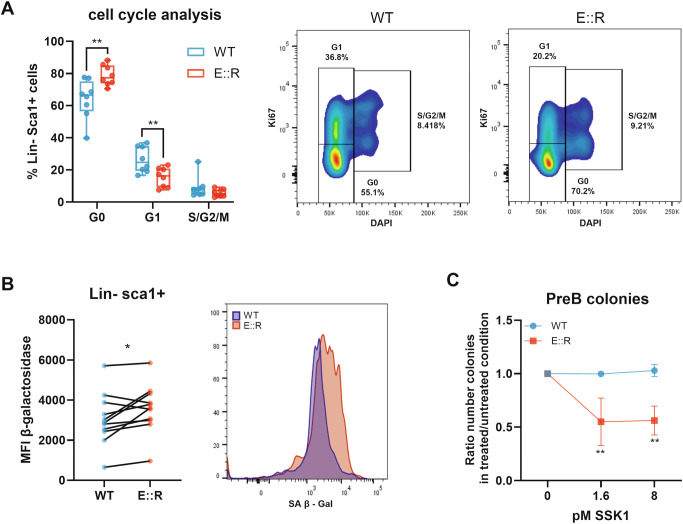
Identification and targeting of OIS markers in Lin^−^Sca1^+^ cells. **A** On the left, bar plots show the percentage of Lin- Sca1+ cells in G0, G1 and S/G2/M phases. On the right, representative flow cytometry analysis of Lin- Sca1+ cells in G0, G1 and S-G2-M, stained with DAPI and anti-Ki67 antibody, from WT (*n* = 8) and E::R mice (*n* = 8). Welch’s *t*-tests, a correction for multiple comparisons with the false discovery rate (FDR = 5%) approach using the Benjamini, Krieger and Yekutieli method has been performed **p < 0.01. **B** On the left, the graph represents the MFI quantification of the β Gal activity detected in WT and Sca1-E::R Lin- Sca1+ population in eleven independent experiments (15 miceper group in total). On the right, representative flow cytometry analysis of β Gal activity in WT and Sca1-E::R Lin- Sca1+ population. Student’s paired *t*-test, *p < 0.05. **C** The curve represents the mean (±SD) ratio of the number of pre-B colonies derived from Lin- cells from E::R transgenic or WT mice treated with SSK1 compared to the untreated conditions in five independent experiments. A mix of 2 mice for group has been used in each experiment. One sample *t*-test was performed vs their own untreated. A correction for multiple comparisons with the false discovery rate (FDR = 5%) approach using the Benjamini, Krieger and Yekutieli method was applied; **q < 0.01.

By flow cytometric cell-cycle analysis, we observed that Lin- Sca1+ progenitors from the bone marrow of E::R transgenic mice displayed an increased proportion of cells in G0 phase (% G0 cells: ctr = 64 ± 12.4; E::R = 79 ± 6.3, *q* < 0.01) and a concomitant reduction in the G1 phase (% G1 cells: ctr = 26 ± 8, *q* < 0.01), while S/G2/M phase remained similar (Fig. 6A). Moreover, E::R Lin- Sca1+ progenitor cells showed enhanced SA β-Gal activity compared to WT mice (SA β-Gal MFI: ctr = 1941 ± 939.8; E::R = 2764 ± 1108, *p* = 0.03, Fig. 6B).

To investigate whether the observed increase in SA β-Gal activity in pre-leukemic progenitors made them more sensitive to the senolytic SSK1, we performed a B colony formation assay. After 7 days of culture in methylcellulose medium supplemented with IL-7, we observed a statistically significant reduction of E::R+ Lin- cells-derived pre-B colonies when treated with SSK1 (Ratio E::R pre-B colonies treated *versus* untreated: 1.6 pM SSK1 = 0.57 ± 0.16, q < 0.01; 8 pM SSK1 = 0.53 ± 0.19, q < 0.01). In contrast, the number of pre-B colonies derived from WT Lin- cells remained unchanged (Fig. 6C).

## Discussion

The leukemia-driving E::R fusion gene arises in utero and generates a clinically silent pre-leukemic progenitor that persists in the organism for many years, increasing the susceptibility to leukemic transformation after the accumulation of additional mutations. These pre-leukemic cells represent the reservoir for the clonal progression to relapse.

Therefore, gaining insights into the molecular mechanisms underlying the survival of E::R+ pre-leukemic cells and their progression to full-blown leukemia will enable the development of novel therapeutic strategies aimed at completely eradicating resistant pre-leukemic cells and, more ambitiously, preventing disease onset.

In this study, we investigated the potential of the E::R fusion gene to induce OIS, a mechanism previously reported to characterize the premalignant lesions of various solid tumors, including melanoma, colon and lung adenomas and prostatic intraepithelial neoplasia [48]. This mechanism was also found to be implicated in hematopoietic tumors, in particular in the development of myelodysplastic syndromes and their progression to AML [49].

Our findings revealed that E::R expression in pro-B BaF3 cells induces hallmark features of senescence, including altered cell morphology, increased SA β-Gal activity, and elevated ROS production. We also found that E::R expression enhances the secretion of various cytokines and chemokines, most of which are pro-inflammatory factors, contributing to the SASP profile of E::R-expressing pro-B cells. Among these factors, CCL2 (also named MCP-1), CXCL2 (also named GRO β), and PAI-1 are frequently detected in the SASP profile of senescent cells [29, 30]. PAI-1 was observed to be increased also in lysates from E::R+ cells. Notably, its intracellular form is implicated in the inhibition of apoptosis through the suppression of caspase-3 activity [47]. Moreover, CCL7, CCL11, and CXCL5 (also named MCP-3, EOTAXIN, and ENA78 respectively) are commonly hyper-secreted specifically in OIS [29].

These changes were accompanied by significant alterations in the gene expression profiles of E::R-expressing cells, particularly in genes involved in p53 pathway and implicated in the induction and maintenance of the senescence response. Significantly, we observed overexpression of genes that inhibit cell cycle progression and dysregulation of genes regulating p53 and p38 MAP kinase pathways. Regarding these, we detected an accumulation of both total p53 protein and the phosphorylated form of p38 MAPK (pp38-Thr180/Tyr182) in E::R+ cells. Similar findings have been reported in human primary fibroblasts upon OIS induced by RUNX1::ETO [21, 38]. In particular, Wolyniec and colleagues suggested a mechanistic link between elevated ROS levels, phosphorylated p38 MAPK, and p53 accumulation in promoting senescence in RUNX1::ETO+ cells [38]. In a related study, Anderson et al. analyzed the SASP associated with RUNX1::ETO-induced OIS and reported upregulation of CCL2, CCL7, and CCL11, suggesting shared features between RUNX1::ETO- and E::R-induced OIS [21]. However, these studies did not observe the ability of E::R to induce a senescent phenotype in human primary fibroblasts [21, 38], highlighting the importance of cellular context in determining the senescence-inducing potential of an oncogene [50].

Interestingly, in line with recent data from Wray and colleagues [12], we observed that despite the accumulation of p53 protein and activation of its cell cycle arrest-associated target genes, there were no changes in the expression of its DNA damage-responsive pro-apoptotic targets in pro-B BaF3 cells.

To further investigate p53 alterations, we analyzed its post-translational modifications. Under genotoxic stress, E::R-expressing cells did not undergo apoptosis and lacked phosphorylation at serine 392 on p53, which promotes p53 mitochondrial translocation and therefore apoptosis [41]. The absence of this phosphorylation may be at least partially attributable to the downregulation of CKIIα kinase activity, although additional studies are necessary to elucidate the underlying mechanism. In contrast, other post-translational modifications, such as phosphorylation at serine 15 (pp53-S15), were unaffected.

Together, these results support a model in which, during the pre-leukemic phase, E::R does not simply trigger a tumor-suppressive OIS program, but rather reconfigures the senescence response into a pro-survival state. In this model, E::R activates upstream components of the senescence machinery, such as p53 accumulation, cell-cycle arrest, and increased phosphorylation of histone H2AX, but selectively uncouples them from the downstream execution of apoptosis. These observations suggest that E::R actively redirects p53 signaling toward a senescence-associated program dominated by cell-cycle inhibition rather than an apoptotic outcome.

In this context, the senescence-like state induced by E::R does not represent a terminal fate but rather an early checkpoint. It allows the persistence of a pool of quiescent, stress-resistant cells that are not fully transformed but remain primed for leukemic progression. Moreover, the increased secretion of pro-inflammatory factors and the resistance to apoptosis in response to genotoxic stress may play a crucial role in facilitating slow clonal expansion and increasing the probability of acquiring additional genetic alterations required to escape senescence and re-enter a proliferative state, ultimately leading to full leukemic transformation, a rare event estimated to occur in approximately 1% of children under the age of 15 carrying E::R [5, 6].

Thus, E::R-induced senescence does not act as a barrier to leukemic transformation. Instead, it establishes a pre-leukemic checkpoint, a state in which cells temporarily arrest proliferation and resist genotoxic stress. This model illustrates how oncogene-induced senescence, rather than suppressing tumorigenesis, can paradoxically promote leukemogenesis.

To selectively eliminate pre-leukemic cells, we employed senolytic drugs targeting multiple key features of the E::R+ pre-leukemic senescence phenotype. Specifically, we successfully targeted increased SA-β-Gal activity, elevated ROS levels, and upregulated PAI-1 using SSK1, PL, and TM5441, respectively.

Remarkably, PL is a non-toxic dietary natural product, derived from long pepper plants, already known for its antitumor properties, although its mechanism of action is not completely elucidated [51]. Importantly, PL does not affect healthy B cells [52], which makes it a highly promising candidate not only for leukemia treatment but also for the prevention of the disease through targeting of pre-leukemic cells.

Lastly, we showed that E::R+Lin- Sca1+ progenitor cells, derived from the Sca1-E::R transgenic mice, exhibited an accumulation in the G0 cell cycle phase, increased SA β-Gal activity and sensitivity to the SSK1 senolytic drug, which significantly reduced the formation of Sca1-E::R+ pre-B cell colonies. These results further corroborate the observations made in BaF3 pro-B cells and highlight the potential of senolytic drugs to eradicate E::R+ pre-leukemic cells, although future in vivo studies and investigations in human models will be essential to validate these results and to strengthen the translational relevance of our observations.

In conclusion, our study highlights the ability of E::R to promote an OIS response in pre-leukemic cells, providing new insights into the mechanisms underlying pre-leukemic cell resistance and progression to leukemia. By elucidating the molecular and cellular features of senescence in this context, we identified potential actionable therapeutic vulnerabilities that may be targeted using senolytic drugs.

These findings pave the way for future studies aimed to develop innovative strategies to eradicate pre-leukemic cells, potentially reducing relapse rates and possibly increasing the chances of preventing the disease onset.

## Materials and methods

### E::R-inducible BaF3 cell model

The BaF3 E::R-expressing model (a gift of Dr A.M. Ford, Centre for Evolution and Cancer, The Institute of Cancer Research, London, UK) is an inducible system in which the expression of E::R is controlled by the supplementation of mifepristone into the cell culture medium (Advanced RPMI 1640 supplemented with 10% fetal bovine serum). The mifepristone-inducible GeneSwitch system (Invitrogen, Waltham, USA) was used to express E::R fused to the V5 epitope as previously described [11].

### Sca1-ETV6-RUNX1 pre-leukemic mouse model

Transgenic Sca1-ETV6-RUNX1 mice [28] and wild-type (WT) siblings, aged 8–14 weeks, were used in the study. Both male and female mice were included. To preserve the study’s focus on the pre-leukemic phase, animals were housed in a specific-pathogen-free facility to prevent external stimuli that could induce mutational accumulation and promote progression toward full leukemic transformation [28]. Transgenic mice carrying the *E::R* sequence were identified via PCR. The primer sequences used for the amplification of *E::R* were 5’-GCCCCATGGAGAATAATCACTG-3’ and 5’-GCGTCTCTAGAAGGATTCATTC-3’.

### Flow cytometry analysis

To evaluate E::R expression in BaF3 inducible model, control and E::R+ BaF3 cells were treated with mifepristone ranging 5-10 pM (Invitrogen) and E::R expression efficiency was assessed by flow-cytometry using BD FacsCANTO II with FITC-conjugated anti-V5 antibody (#Ab1274, Abcam, Cambridge, UK) before each experiment. Only when E::R expression efficiency was > 80%, cells were used. To detect cellular ROS levels, cells were incubated with 5 μg of CM-H2DCFDA probe (Invitrogen) at 37 °C for 30 min and its median fluorescence intensity (MFI) was measured. For the competitive assay, E::R+ and control BaF3 cells were mixed at a starting ratio of 80:20 and cultured with and without different concentrations of etoposide or doses of x-ray. After 48 h, the percentage of E::R+ BaF3 cells was analyzed using the anti-V5 tag antibody. To perform the viability assay, the cells were treated with vehicle or etoposide (Selleckchem, Houston, USA), SSK1 (MedChemExpress, Monmouth Junction, USA), piperlongumine (MedChemExpress) or TM5441 (Tocris, Bristol, UK) for 2 days. After the treatment, cells were harvested and stained with Annexin V-PE (Enzo Life Science, Farmingdale, USA) following the manufacturer’s instructions. For the cell cycle analysis in Lin- Sca1+ cells from WT and E::R mice, total bone marrow cells were isolated and enriched for Lin- cell population using Direct Cell Lineage Depletion kit (Miltenyi, Cologne, Germany) according to the Manufacturer’s instruction. Then, Lin- cells were stained with Sca1-APC antibody (eBioscience, clone D7) for 30 min at 4 °C, permeabilized 20 min at 4 °C with Citofix/Cytoperm kit (BD Biosciences, New Jersey, USA), washed and stained with Ki67-FITC (eBioscience, clone SolA15) 30 min at 4 °C. Immediately before acquisition, cells were washed and stained with 1 µg/ml of DAPI (Merck, Darmstadt, DE). For the flow cytometric analysis of senescence-associated β-Galactosidase (SA β-Gal) activity, we used the Senescence assay Kit (ab228562, Abcam) according to the manufacturer’s instructions. Briefly, after Lin- enrichment, the cells were incubated for 2 h with the Senescence Dye at 37 °C in IMDM medium containing 2% serum. Then, the cells were stained for Sca1 antigen and analyzed.

### Immunofluorescence analysis

The immunofluorescence analysis was performed as previously described [23]. Briefly, cells grown on polylysine-coated coverslips were fixed with 4% paraformaldehyde. The cytoskeletal actin was labeled with Alexa Fluor 546 phalloidin (Invitrogen). Confocal microscopy was carried out on a Radiance 2100 microscope (Bio-Rad, Hercules, USA) equipped with a Kripon/Argon laser.

### Colorimetric SA β-GAL activity analysis

We used the Senescence β-Galactosidase Staining Kit (#9860, Cell Signaling, Danvers, USA) to colorimetrically detect the SA β-Gal activity according to the manufacturer’s instructions. Briefly, the control and E::R cells were cultured with the inducer for 2 days. The cells were fixed, stained with the β-Galactosidase Staining Solution and incubated overnight at 37 °C without CO_2_. At the end of the assay, we took pictures of the cells with an optical microscope (10× objective) and analyzed them using ImageJ software to quantify the number of total cells and blue cells to obtain the percentage of blue-positive cells.

### RNA expression analysis

The gene expression profile experiment was performed as previously reported [23]. Gene Set Enrichment Analysis (GSEA) was performed using GSEA version 4.1.0, with genes ranked by signal-to-noise ratio and statistically significant gene sets determined by 1000 permutations and geneset permutations. For GSEA, the false discovery rate (FDR) cutoff < 0.05 and a minimum gene set size of 15 were applied for the analysis. The probe sets were collapsed to genes for max probe. MSigDB c2CGP and Hallmark of Mouse gene sets in MSigDB (v.2024.1). ClusterProfiler (v4.14.6) package in R BioConductor was used to perform GSEA plot by gseaplot2 function [53]. Pheatmap package (v.1.0.12) was used to generate heatmap.

### Western blot analysis

After culturing E::R+ and control BaF3 cells with mifepristone for 2 days, proteins were extracted with NP40 lysis buffer (Invitrogen) supplemented with 1:100 dilution of protease inhibitor cocktail (Sigma, Burlington, Massachusetts) and 0.25 mM of PMSF (Sigma). For the analysis of pp53-ser15 and pp53-ser392, both populations were treated with 0.75 or 1 µg/ml of etoposide (Selleckchem) for different time points. For the analysis of pp53-ser392 in only control cells, 0.5 µg/ml of etoposide (Selleckchem) was used in combination with 5 and 10 µM of silmitasertib (Aurogene, Rome, Italy) for 2 days. Protein concentration was determined using the BCA Protein Assay (Invitrogen). 25 μg of proteins were separated on 4-12% Bis-Tris gels (Invitrogen) and transferred to a PVDF membrane. According to each antibody-specific protocol provided by the manufacturer, the membrane was blocked with 5% non-fat dry milk or 5% BSA in TBST 1X for at least 1 h and half. The membrane was incubated with the primary antibody at 4 °C overnight, washed three times with TBST, and incubated for 1 h with the appropriate secondary antibody. The proteins were detected using the Alliance Q9 Advanced (Uvitec, Cambridge, UK). The following antibodies were used: mouse anti-p53 (1:1000, #2524S, Cell Signaling,), mouse anti-pp53-S15 (1:1000, #9284S, Cell Signaling), mouse anti-pp53-S392 (1:1000, #9281S, Cell Signaling), mouse anti-CKII alpha (1:1000, #2656S, Cell Signaling), mouse anti-p38 (1:1000, #9212S, Cell Signaling), mouse anti-pp38 (1:1000, #4511S, Cell Signaling), mouse anti-actin Ab (1:6000, #A1978, Merck), goat anti-mouse IgG (1:20000, #A9309, Merck).

### Cytokines and chemokines quantification

Control and E::R cells were cultured for 2 days in the presence of mifepristone to induce the expression of the fusion gene. Subsequently, they were maintained for an additional day in medium supplemented with 5% fetal bovine serum, after which the conditioned medium was collected. The conditioned media were centrifuged at 10000 × *g* for 10 min and the resulting supernatants were stored at −80 °C until use. Cytokines and chemokines concentrations were determined using a ProcartaPlex Mouse Cytokines/Chemokines Magnetic Bead Panel (EPX360-26092-901, Invitrogen) (Supplementary Table S1) with Luminex MAGPIX (Sigma) according to manufacturer’s instructions. Cytokines/Chemokines below the level of detectability in both control and E::R-conditioned media samples were excluded from the analysis. Plasminogen activator inhibitor-1 (PAI-1) quantification in E::R and control-conditioned media and lysates was performed using ELISA kit (R&D, Minneapolis, USA) according to the manufacturer’s manual. Lysates were obtained using NP40 lysis buffer (Invitrogen) supplemented with 1:100 dilution of protease inhibitor cocktail (Sigma) and 0.25 mM of PMSF (Sigma).

The concentration of the secreted cytokines/chemokines was normalized to the total number of cells. The protein concentration of the lysates was determined using the BCA Protein Assay (Invitrogen) and 40 μg were used for the intracellular quantification of PAI-1.

When a value was below the detectable limit, its concentration was defined as half of the lowest standard concentration.

### Colony-forming unit assays

Total bone marrow cells were isolated from WT and Sca1-ETV6-RUNX1 mice. Samples were enriched for Lin- population using Direct Cell Lineage Depletion kit (Miltenyi) according to the Manufacturer’s instruction. Then, Lin- cells were seeded in 1.1 ml duplicate methylcellulose cultures in 6-well dishes (3 ×10^3^ cells/well). Methocult M3630 (Stem Cell Technologies, Vancouver, Canada) containing IL-7, SCF and FLT3L was used to assess the ability to generate pre-B lymphoid progenitor cells in vitro. Cells were treated with vehicle or SSK1 at 1.6 and 8 pM. After 7 days, the number of pre-B colonies was scored.

### Statistical analyses

Each experiment was conducted in at least five independent replicates and their mean was considered for the analyses. Data were described using relative or absolute frequencies or mean and standard deviation (SD), as appropriate. Comparisons between a group with a reference value or between two dependent/independent groups were performed by means of a Student’s *t* test for one or two independent/paired samples. Multiple comparisons were analyzed using a false discovery rate (FDR) correction at 5%, applied according to the Benjamini, Krieger, and Yekutieli method. Statistical significance was set at 0.05. All statistical analyses were conducted using Prism 9.4 version.

## Supplementary information

Supplementary table S1

Supplementary table S2

Supplementary table S3

Supplementary information

Uncropped western blot images

## Data Availability

The datasets generated and/or analyzed during the current study are available in the Gene Expression Omnibus repository (https://www.ncbi.nlm.nih.gov/geo/query/acc.cgi?acc=GSE134437).
