# Supplementary material for: Targeting oncogene-induced senescence in ETV6::RUNX1 pre-leukemic cells
Source: Cell Death Discov. 2026 Mar 11;12:145. doi: 10.1038/s41420-026-03001-5 (PMC13039127; doi:10.1038/s41420-026-03001-5)
Supplement: Supplementary file 1 — Supplementary table S1 [file 41420_2026_3001_MOESM1_ESM.pdf]

Supplementary Table S1. List of the cytokines and chemokines analyzed with ProcartaPlex Mouse Cytokines/Chemokines panel.

Cytokines/Chemokines					
CCL2	CCL3	CCL4	CCL5	CCL7	CXCL2
ENA-78	EOTAXIN	G-CSF	GM-CSF	GROa	IL-10
IL-12p70	IL-13	IL-15	IL-17a	IL-18	IL-1a
IL-1b	IL-2	IL-22	IL-23	IL-27	IL-28
IL-3	IL-31	IL-4	IL-5	IL-6	IL-9
INFa	INFg	IP-10	LIF	M-CSF	TNFa
