# Supplementary material for: Targeting oncogene-induced senescence in ETV6::RUNX1 pre-leukemic cells
Source: Cell Death Discov. 2026 Mar 11;12:145. doi: 10.1038/s41420-026-03001-5 (PMC13039127; doi:10.1038/s41420-026-03001-5)
Supplement: Supplementary file 2 — Supplementary table S2 [file 41420_2026_3001_MOESM2_ESM.pdf]

Supplementary Table S2. Table of ONGUSAHA_TP53_TARGETS gene set: gene description, ranking, individual enrichment scores, core enrichment Yes/No.

GENE_SYMBOL	GENE_TITLE	RANK IN GENE LIST	RANK METRIC SCORE	RUNNING ES	CORE ENRICHMENT
Fas	Fas cell surface death receptor [Source:MGI Symbol;Acc:MGI:95484]	195	0.695139467716217	0.084022544	Yes
Ak1	adenylate kinase 1 [Source:MGI Symbol;Acc:MGI:87977]	331	0.5292492508888245	0.14868571	Yes
Zmat3	zinc finger matrin type 3 [Source:MGI Symbol;Acc:MGI:1195270]	332	0.5287314057350159	0.2202172	Yes
Ctsh	cathepsin H [Source:MGI Symbol;Acc:MGI:107285]	338	0.5243664979934692	0.29090118	Yes
Tob1	transducer of ErbB-2.1 [Source:MGI Symbol;Acc:MGI:1349721]	571	0.3766686022281647	0.32993653	Yes
Phlda3	pleckstrin homology like domain, family A, member 3 [Source:MGI Symbol;Acc:MGI:1351485]	594	0.3658870458602905	0.37830627	Yes
Ddit4	DNA-damage-inducible transcript 4 [Source:MGI Symbol;Acc:MGI:1921997]	643	0.3477589190006256	0.4228872	Yes
Nqo1	NAD(P)H dehydrogenase, quinone 1 [Source:MGI Symbol;Acc:MGI:103187]	658	0.3441297113895416	0.4687246	Yes
Lpin1	lipin 1 [Source:MGI Symbol;Acc:MGI:1891340]	664	0.3430095314979553	0.514873	Yes
Pltp	phospholipid transfer protein [Source:MGI Symbol;Acc:MGI:103151]	681	0.3344212770462036	0.55929416	Yes
Ercc5	excision repair cross-complementing rodent repair deficiency, complementation group 5 [Source:MGI Symbol;Acc:MGI:103582]	879	0.27609798312187195	0.5865223	Yes
Mdm2	transformed mouse 3T3 cell double minute 2 [Source:MGI Symbol;Acc:MGI:96952]	886	0.2748461663722992	0.6233975	Yes
Ptprv	protein tyrosine phosphatase receptor type V [Source:MGI Symbol;Acc:MGI:108027]	994	0.253793865442276	0.6522337	Yes
Sat1	spermidine/spermine N1-acetyl transferase 1 [Source:MGI Symbol;Acc:MGI:98233]	1063	0.24013520777225494	0.68122643	Yes
Apobec1	apolipoprotein B mRNA editing enzyme, catalytic polypeptide 1 [Source:MGI Symbol;Acc:MGI:103298]	1084	0.23560023307800293	0.7120726	Yes
Cdkn1a	cyclin dependent kinase inhibitor 1A [Source:MGI Symbol;Acc:MGI:104556]	1085	0.2354511171579361	0.7439265	Yes
Ccng1	cyclin G1 [Source:MGI Symbol;Acc:MGI:102890]	1372	0.19993899762630463	0.75627697	Yes
Slc66a3	solute carrier family 66 member 3 [Source:MGI Symbol;Acc:MGI:2444067]	1748	0.162441685795784	0.7589803	Yes
Ivl	involucrin [Source:MGI Symbol;Acc:MGI:96626]	2121	0.13748770952224731	0.7584618	Yes
Pitpnc1	phosphatidylinositol transfer protein, cytoplasmic 1 [Source:MGI Symbol;Acc:MGI:1919045]	2266	0.1286478489637375	0.76846546	Yes
Fxyd3	FXYD domain-containing ion transport regulator 3 [Source:MGI Symbol;Acc:MGI:107497]	3639	0.07429797947406769	0.7080027	No
Dgka	diacylglycerol kinase, alpha [Source:MGI Symbol;Acc:MGI:102952]	4511	0.052788134664297104	0.6703789	No
Ctsf	cathepsin F [Source:MGI Symbol;Acc:MGI:1861434]	4600	0.05086047947406769	0.672737	No
Srxn1	sulfiredoxin 1 homolog (S. cerevisiae) [Source:MGI Symbol;Acc:MGI:104971]	5375	0.035473305732011795	0.6377561	No
Serpine2	serine (or cysteine) peptidase inhibitor, clade E, member 2 [Source:MGI Symbol;Acc:MGI:101780]	6115	0.022395644336938858	0.6028048	No
Enpp2	ectonucleotide pyrophosphatase/phosphodiesterase 2 [Source:MGI Symbol;Acc:MGI:1321390]	7057	0.008237048052251339	0.5555561	No
Daxx	Fas death domain-associated protein [Source:MGI Symbol;Acc:MGI:1197015]	7653	3,1911670248518900E+09	0.5249763	No
Robo1	roundabout guidance receptor 1 [Source:MGI Symbol;Acc:MGI:1274781]	8030	-0.004700896330177784	0.50628763	No
Tap1	transporter 1, ATP-binding cassette, sub-family B (MDR/TAP) [Source:MGI Symbol;Acc:MGI:98483]	9244	-0.01942824386060238	0.44657344	No
Matn4	matrilin 4 [Source:MGI Symbol;Acc:MGI:1328314]	9350	-0.02060389518737793	0.4439644	No
Tnfrsf18	tumor necrosis factor receptor superfamily, member 18 [Source:MGI Symbol;Acc:MGI:894675]	9465	-0.022151384502649307	0.44110215	No
Ly6a	lymphocyte antigen 6 family member A [Source:MGI Symbol;Acc:MGI:107527]	10795	-0.03798067569732666	0.37793607	No
Trp53	transformation related protein 53 [Source:MGI Symbol;Acc:MGI:98834]	11026	-0.04079430177807808	0.37163413	No
Ephx1	epoxide hydrolase 1, microsomal [Source:MGI Symbol;Acc:MGI:95405]	11205	-0.04283653572201729	0.36828107	No
Efs	embryonal Fyn-associated substrate [Source:MGI Symbol;Acc:MGI:105311]	13552	-0.07314013689756393	0.25760254	No
Col18a1	collagen, type XVIII, alpha 1 [Source:MGI Symbol;Acc:MGI:88451]	15408	-0.10347612202167511	0.17626327	No
Anxa8	annexin A8 [Source:MGI Symbol;Acc:MGI:1201374]	15655	-0.10844932496547699	0.17829199	No
Csrp2	cysteine and glycine-rich protein 2 [Source:MGI Symbol;Acc:MGI:1202907]	16998	-0.1401711404323578	0.12828298	No
