# Supplementary material for: Targeting oncogene-induced senescence in ETV6::RUNX1 pre-leukemic cells
Source: Cell Death Discov. 2026 Mar 11;12:145. doi: 10.1038/s41420-026-03001-5 (PMC13039127; doi:10.1038/s41420-026-03001-5)
Supplement: Supplementary file 3 — Supplementary table S3 [file 41420_2026_3001_MOESM3_ESM.pdf]

Supplementary Table S3 Table of HALLMARK_P53_PATHWAY gene set: gene description, ranking, individual enrichment scores, core enrichment Yes/No.

GENE_SYMBOL	GENE_TITLE	RANK IN GENE LIST	RANK METRIC SCORE	RUNNING ES	CORE ENRICHMENT
Slc7a11	solute carrier family 7 (cationic amino acid transporter, y+ system), member 11 [Source:MGI Symbol;Acc:MGI:1347355]	106	0.9255611300468445	0.025571374	Yes
Fas	Fas cell surface death receptor [Source:MGI Symbol;Acc:MGI:95484]	195	0.695139467716217	0.044341955	Yes
Gadd45a	growth arrest and DNA-damage-inducible 45 alpha [Source:MGI Symbol;Acc:MGI:107799]	206	0.6769422292709351	0.06654366	Yes
Trib3	tribbles pseudokinase 3 [Source:MGI Symbol;Acc:MGI:1345675]	229	0.6375668048858643	0.086802	Yes
Notch1	notch 1 [Source:MGI Symbol;Acc:MGI:97363]	253	0.6015861630439758	0.10580092	Yes
Ak1	adenylate kinase 1 [Source:MGI Symbol;Acc:MGI:87977]	331	0.5292492508888245	0.11957381	Yes
Zmat3	zinc finger matrin type 3 [Source:MGI Symbol;Acc:MGI:1195270]	332	0.5287314057350159	0.13731937	Yes
Rrad	Ras-related associated with diabetes [Source:MGI Symbol;Acc:MGI:1930943]	353	0.5061187148094177	0.15326962	Yes
Dram1	DNA-damage regulated autophagy modulator 1 [Source:MGI Symbol;Acc:MGI:1918962]	369	0.49967336654663086	0.16926263	Yes
Nupr1	nuclear protein transcription regulator 1 [Source:MGI Symbol;Acc:MGI:1891834]	428	0.44812020659446716	0.18129718	Yes
Tcn2	transcobalamin 2 [Source:MGI Symbol;Acc:MGI:98534]	434	0.4437083899974823	0.19593006	Yes
Itgb4	integrin beta 4 [Source:MGI Symbol;Acc:MGI:96613]	445	0.4369165897369385	0.2100759	Yes
Btg2	BTG anti-proliferation factor 2 [Source:MGI Symbol;Acc:MGI:108384]	446	0.43575626611709595	0.22470099	Yes
Casp1	caspase 1 [Source:MGI Symbol;Acc:MGI:96544]	499	0.40768739581108093	0.23568943	Yes
Acvr1b	activin A receptor, type 1B [Source:MGI Symbol;Acc:MGI:1338944]	545	0.388302743434906	0.24639	Yes
Tob1	transducer of ErbB-2.1 [Source:MGI Symbol;Acc:MGI:1349721]	571	0.3766686022281647	0.25773647	Yes
Zbtb16	zinc finger and BTB domain containing 16 [Source:MGI Symbol;Acc:MGI:103222]	579	0.3732067048549652	0.26989952	Yes
Phlda3	pleckstrin homology like domain, family A, member 3 [Source:MGI Symbol;Acc:MGI:1351485]	594	0.3658870458602905	0.28145415	Yes
Cdkn2b	cyclin dependent kinase inhibitor 2B [Source:MGI Symbol;Acc:MGI:104737]	597	0.36543571949005127	0.29361546	Yes
Cd81	CD81 antigen [Source:MGI Symbol;Acc:MGI:1096398]	604	0.36437758803367615	0.30553398	Yes
Ifi30	interferon gamma inducible protein 30 [Source:MGI Symbol;Acc:MGI:2137648]	614	0.3610899746417999	0.31718668	Yes
Cd82	CD82 antigen [Source:MGI Symbol;Acc:MGI:104651]	632	0.3513374328613281	0.32809755	Yes
Rap2b	RAP2B, member of RAS oncogene family [Source:MGI Symbol;Acc:MGI:1921262]	633	0.3512916564941406	0.33988777	Yes
Ddit4	DNA-damage-inducible transcript 4 [Source:MGI Symbol;Acc:MGI:1921997]	643	0.3477589190006256	0.35109308	Yes
Vwa5a	von Willebrand factor A domain containing 5A [Source:MGI Symbol;Acc:MGI:1915026]	849	0.2822255790233612	0.34994242	Yes
Ercc5	excision repair cross-complementing rodent repair deficiency, complementation group 5 [Source:MGI Symbol;Acc:MGI:103582]	879	0.27609798312187195	0.35770622	Yes
Mdm2	transformed mouse 3T3 cell double minute 2 [Source:MGI Symbol;Acc:MGI:96952]	886	0.2748461663722992	0.36661983	Yes
Csrnp2	cysteine-serine-rich nuclear protein 2 [Source:MGI Symbol;Acc:MGI:2386852]	893	0.2725428342819214	0.37545615	Yes
Ada	adenosine deaminase [Source:MGI Symbol;Acc:MGI:87916]	945	0.2615340054035187	0.38159114	Yes
Ndrg1	N-myc downstream regulated gene 1 [Source:MGI Symbol;Acc:MGI:1341799]	953	0.2603650391101837	0.3899669	Yes
Sat1	spermidine/spermine N1-acetyl transferase 1 [Source:MGI Symbol;Acc:MGI:98233]	1063	0.24013520777225494	0.3923782	Yes
Cdkn1a	cyclin dependent kinase inhibitor 1A [Source:MGI Symbol;Acc:MGI:104556]	1085	0.2354511171579361	0.39919233	Yes
Stom	stomatin [Source:MGI Symbol;Acc:MGI:95403]	1115	0.23166625201702118	0.4054649	Yes
Ddit3	DNA-damage inducible transcript 3 [Source:MGI Symbol;Acc:MGI:109247]	1323	0.20467112958431244	0.4016077	Yes
Ccng1	cyclin G1 [Source:MGI Symbol;Acc:MGI:102890]	1372	0.19993899762630463	0.40583083	Yes
Ier5	immediate early response 5 [Source:MGI Symbol;Acc:MGI:1337072]	1377	0.19952140748500824	0.41232	Yes
Btg1	BTG anti-proliferation factor 1 [Source:MGI Symbol;Acc:MGI:88215]	1425	0.19393464922904968	0.41639346	Yes
Mxd1	MAX dimerization protein 1 [Source:MGI Symbol;Acc:MGI:96908]	1440	0.1925080120563507	0.42212903	Yes
Socs1	suppressor of cytokine signaling 1 [Source:MGI Symbol;Acc:MGI:1354910]	1493	0.18625237047672272	0.42568555	Yes
Plk3	polo like kinase 3 [Source:MGI Symbol;Acc:MGI:109604]	1575	0.17818081378936768	0.42746845	Yes
Rad51c	RAD51 paralog C [Source:MGI Symbol;Acc:MGI:2150020]	1608	0.17526306211948395	0.4316925	Yes
Slc19a2	solute carrier family 19 (thiamine transporter), member 2 [Source:MGI Symbol;Acc:MGI:1928761]	1672	0.16982944309711456	0.43412784	Yes
Slc35d1	solute carrier family 35 (UDP-glucuronic acid/UDP-N-acetylgalactosamine dual transporter), member D1 [Source:MGI Symbol;Acc:MGI:2140361]	1695	0.167241632938385	0.43860087	Yes
Eps8l2	EPS8-like 2 [Source:MGI Symbol;Acc:MGI:2138828]	1697	0.16702692210674286	0.4441549	Yes
Elp1	elongator complex protein 1 [Source:MGI Symbol;Acc:MGI:1914544]	1704	0.16574014723300934	0.44940662	Yes
Alox8	arachidonate 8-lipoxygenase [Source:MGI Symbol;Acc:MGI:1098228]	1738	0.16325749456882477	0.45317593	Yes
Ptpn14	protein tyrosine phosphatase, non-receptor type 14 [Source:MGI Symbol;Acc:MGI:102467]	1742	0.1630582958459854	0.45849314	Yes

Ccnd3	cyclin D3 [Source:MGI Symbol;Acc:MGI:88315]	1891	0.15225258469581604	0.45593393	Yes
Abhd4	abhydrolase domain containing 4 [Source:MGI Symbol;Acc:MGI:1915938]	1904	0.15168289840221405	0.46040297	Yes
Fuca1	fucosidase, alpha-L- 1, tissue [Source:MGI Symbol;Acc:MGI:95593]	1910	0.15121138095855713	0.4652189	Yes
Jun	jun proto-oncogene [Source:MGI Symbol;Acc:MGI:96646]	2003	0.14398179948329926	0.46528396	Yes
Rb1	RB transcriptional corepressor 1 [Source:MGI Symbol;Acc:MGI:97874]	2091	0.13928206264972687	0.46545038	Yes
Pvt1	Pvt1 oncogene [Source:MGI Symbol;Acc:MGI:97824]	2099	0.13878464698791504	0.4697456	Yes
Trafd1	TRAF type zinc finger domain containing 1 [Source:MGI Symbol;Acc:MGI:1923551]	2171	0.13386376202106476	0.4705593	Yes
Nudt15	nudix hydrolase 15 [Source:MGI Symbol;Acc:MGI:2443366]	2235	0.13037331402301788	0.47167036	Yes
Pitpnc1	phosphatidylinositol transfer protein, cytoplasmic 1 [Source:MGI Symbol;Acc:MGI:1919045]	2266	0.1286478489637375	0.47443354	Yes
Ptpre	protein tyrosine phosphatase receptor type E [Source:MGI Symbol;Acc:MGI:97813]	2328	0.12513868510723114	0.47547257	Yes
Tgfb1	transforming growth factor, beta 1 [Source:MGI Symbol;Acc:MGI:98725]	2478	0.11708106100559235	0.4716811	Yes
Zfp36l1	zinc finger protein 36, C3H type-like 1 [Source:MGI Symbol;Acc:MGI:107946]	2486	0.11680319905281067	0.47523856	Yes
Gpx2	glutathione peroxidase 2 [Source:MGI Symbol;Acc:MGI:106609]	2493	0.1165495216846466	0.47883934	Yes
Txnip	thioredoxin interacting protein [Source:MGI Symbol;Acc:MGI:1889549]	2538	0.11429238319396973	0.48039526	Yes
Tnfsf9	tumor necrosis factor (ligand) superfamily, member 9 [Source:MGI Symbol;Acc:MGI:1101058]	2601	0.11143004149198532	0.48092237	Yes
Irak1	interleukin-1 receptor-associated kinase 1 [Source:MGI Symbol;Acc:MGI:107420]	2716	0.10628962516784668	0.47858238	Yes
Ei24	etoposide induced 2.4 mRNA [Source:MGI Symbol;Acc:MGI:108090]	2744	0.10502862930297852	0.48070827	Yes
Tax1bp3	Tax1 (human T cell leukemia virus type I) binding protein 3 [Source:MGI Symbol;Acc:MGI:1923531]	2794	0.10295907407999039	0.48162472	Yes
Mxd4	Max dimerization protein 4 [Source:MGI Symbol;Acc:MGI:104991]	2878	0.09944368898868561	0.48066133	Yes
Gm2a	GM2 ganglioside activator protein [Source:MGI Symbol;Acc:MGI:95762]	2887	0.0991358757019043	0.48357403	Yes
Ddb2	damage specific DNA binding protein 2 [Source:MGI Symbol;Acc:MGI:1355314]	2922	0.09747560322284698	0.48508373	Yes
Ip6k2	inositol hexaphosphate kinase 2 [Source:MGI Symbol;Acc:MGI:1923750]	3011	0.09438322484493256	0.48369142	No
Cdk5r1	cyclin dependent kinase 5, regulatory subunit 1 [Source:MGI Symbol;Acc:MGI:101764]	3143	0.08975686132907867	0.47991562	No
Ankra2	ankyrin repeat family A member 2 [Source:MGI Symbol;Acc:MGI:1915808]	3170	0.08900456130504608	0.48155555	No
Ctsd	cathepsin D [Source:MGI Symbol;Acc:MGI:88562]	3637	0.0743371769785881	0.4599029	No
Baiap2	brain-specific angiogenesis inhibitor 1-associated protein 2 [Source:MGI Symbol;Acc:MGI:2137336]	3668	0.07342775911092758	0.46081275	No
Rab40c	Rab40C, member RAS oncogene family [Source:MGI Symbol;Acc:MGI:2183454]	3715	0.07197104394435883	0.46084464	No
Ppm1d	protein phosphatase 1D magnesium-dependent, delta isoform [Source:MGI Symbol;Acc:MGI:1858214]	4030	0.06379323452711105	0.44671458	No
Fos	FBJ osteosarcoma oncogene [Source:MGI Symbol;Acc:MGI:95574]	4067	0.0626143217086792	0.44695058	No
Rchy1	ring finger and CHY zinc finger domain containing 1 [Source:MGI Symbol;Acc:MGI:1915348]	4278	0.0576348640024662	0.438003	No
Coq8a	coenzyme Q8A [Source:MGI Symbol;Acc:MGI:1914676]	4402	0.054848577827215195	0.43347013	No
Ppp1r15a	protein phosphatase 1, regulatory subunit 15A [Source:MGI Symbol;Acc:MGI:1927072]	4418	0.05457669869065285	0.4345246	No
Dgka	diacylglycerol kinase, alpha [Source:MGI Symbol;Acc:MGI:102952]	4511	0.052788134664297104	0.43152896	No
Pidd1	p53 induced death domain protein 1 [Source:MGI Symbol;Acc:MGI:1889507]	4550	0.051859863102436066	0.4313004	No
Ctsf	cathepsin F [Source:MGI Symbol;Acc:MGI:1861434]	4600	0.05086047947406769	0.43046826	No
Nhlh2	nescient helix loop helix 2 [Source:MGI Symbol;Acc:MGI:97324]	4747	0.04776265472173691	0.42450577	No
Osgin1	oxidative stress induced growth inhibitor 1 [Source:MGI Symbol;Acc:MGI:1919089]	4762	0.04751306399703026	0.42537495	No
Abat	4-aminobutyrate aminotransferase [Source:MGI Symbol;Acc:MGI:2443582]	4800	0.046602632850408554	0.42502177	No
Klk8	kallikrein related-peptidase 8 [Source:MGI Symbol;Acc:MGI:1343327]	4824	0.04619796946644783	0.42538044	No
Fbxw7	F-box and WD-40 domain protein 7 [Source:MGI Symbol;Acc:MGI:1354695]	5119	0.04027409851551056	0.4114974	No
Tchh	trichohyalin [Source:MGI Symbol;Acc:MGI:2177944]	5206	0.03853056952357292	0.40833417	No
Pcna	proliferating cell nuclear antigen [Source:MGI Symbol;Acc:MGI:97503]	5439	0.034385472536087036	0.39746627	No
Prmt2	protein arginine N-methyltransferase 2 [Source:MGI Symbol;Acc:MGI:1316652]	5624	0.031014733016490936	0.38897252	No
Bmp2	bone morphogenetic protein 2 [Source:MGI Symbol;Acc:MGI:88177]	5658	0.030587144196033478	0.3882891	No
Slc3a2	solute carrier family 3 (activators of dibasic and neutral amino acid transport), member 2 [Source:MGI Symbol;Acc:MGI:96955]	5675	0.03020695596933365	0.3884738	No
Triap1	TP53 regulated inhibitor of apoptosis 1 [Source:MGI Symbol;Acc:MGI:1916326]	5810	0.027786443009972572	0.38246268	No
Perp	PERP, TP53 apoptosis effector [Source:MGI Symbol;Acc:MGI:1929938]	5854	0.027124280110001564	0.38114482	No
Tgfa	transforming growth factor alpha [Source:MGI Symbol;Acc:MGI:98724]	5856	0.027085619047284126	0.38200206	No
Jag2	jagged 2 [Source:MGI Symbol;Acc:MGI:1098270]	6122	0.022301439195871353	0.36901855	No
App	amyloid beta precursor protein [Source:MGI Symbol;Acc:MGI:88059]	6157	0.021762888878583908	0.36798713	No
Cdkn2aip	CDKN2A interacting protein [Source:MGI Symbol;Acc:MGI:1918175]	6261	0.019997529685497284	0.36332095	No
Foxo3	forkhead box O3 [Source:MGI Symbol;Acc:MGI:1890081]	6329	0.019067887216806412	0.36048907	No

St14	suppression of tumorigenicity 14 (colon carcinoma) [Source:MGI Symbol;Acc:MGI:1338881]	6358	0.018618183210492134	0.359663	No
Blcap	bladder cancer associated protein [Source:MGI Symbol;Acc:MGI:1858907]	6420	0.01771990954875946	0.3570968	No
Rps27l	ribosomal protein S27-like [Source:MGI Symbol;Acc:MGI:1915191]	6530	0.016018368303775787	0.35198614	No
Procr	protein C receptor, endothelial [Source:MGI Symbol;Acc:MGI:104596]	6556	0.015648143365979195	0.35121587	No
Irag2	inositol 1,4,5-triphosphate receptor associated 2 [Source:MGI Symbol;Acc:MGI:108424]	6871	0.01074548251926899	0.3353054	No
Pmm1	phosphomannomutase 1 [Source:MGI Symbol;Acc:MGI:1353418]	7037	0.008527173660695553	0.32704148	No
Traf4	TNF receptor associated factor 4 [Source:MGI Symbol;Acc:MGI:1202880]	7067	0.008134458214044571	0.32581174	No
H2aj	H2J.A histone [Source:MGI Symbol;Acc:MGI:3606192]	7351	0.003822150407359004	0.3112753	No
Tm7sf3	transmembrane 7 superfamily member 3 [Source:MGI Symbol;Acc:MGI:1914873]	7368	0.003580773016437888	0.31056637	No
Apaf1	apoptotic peptidase activating factor 1 [Source:MGI Symbol;Acc:MGI:1306796]	7378	0.0034407831262797117	0.3102155	No
Abcc5	ATP-binding cassette, sub-family C member 5 [Source:MGI Symbol;Acc:MGI:1351644]	7541	0.0014457268407568336	0.30186936	No
Cdkn2a	cyclin dependent kinase inhibitor 2A [Source:MGI Symbol;Acc:MGI:104738]	7549	0.0012995792785659432	0.30155024	No
Pom121	nuclear pore membrane protein 121 [Source:MGI Symbol;Acc:MGI:2137624]	7876	-0.0028740759007632732	0.28475377	No
Rack1	receptor for activated C kinase 1 [Source:MGI Symbol;Acc:MGI:101849]	7959	-0.0038625705055892467	0.28063425	No
Wwp1	WW domain containing E3 ubiquitin protein ligase 1 [Source:MGI Symbol;Acc:MGI:1861728]	7990	-0.0042289248667657375	0.27922162	No
Rpl36	ribosomal protein L36 [Source:MGI Symbol;Acc:MGI:1860603]	8325	-0.008132413029670715	0.26218706	No
Rps12	ribosomal protein S12 [Source:MGI Symbol;Acc:MGI:98105]	8433	-0.009521747007966042	0.25696203	No
Retsat	retinol saturase (all trans retinol 13,14 reductase) [Source:MGI Symbol;Acc:MGI:1914692]	8656	-0.01222523208707571	0.24586856	No
Hexim1	hexamethylene bis-acetamide inducible 1 [Source:MGI Symbol;Acc:MGI:2385923]	8784	-0.013807862065732479	0.239751	No
Mknk2	MAP kinase-interacting serine/threonine kinase 2 [Source:MGI Symbol;Acc:MGI:894279]	9005	-0.01660529524087906	0.22890817	No
Plxnb2	plexin B2 [Source:MGI Symbol;Acc:MGI:2154239]	9177	-0.018785826861858368	0.22067764	No
Tap1	transporter 1, ATP-binding cassette, sub-family B (MDR/TAP) [Source:MGI Symbol;Acc:MGI:98483]	9244	-0.01942824386060238	0.21790966	No
Kif13b	kinesin family member 13B [Source:MGI Symbol;Acc:MGI:1098265]	9558	-0.023266810923814774	0.20247126	No
Sp1	trans-acting transcription factor 1 [Source:MGI Symbol;Acc:MGI:98372]	9642	-0.024269739165902138	0.19898485	No
Fam162a	family with sequence similarity 162, member A [Source:MGI Symbol;Acc:MGI:1917436]	9652	-0.024359706789255142	0.19933605	No
Hint1	histidine triad nucleotide binding protein 1 [Source:MGI Symbol;Acc:MGI:1321133]	9655	-0.024378977715969086	0.20005062	No
Nol8	nucleolar protein 8 [Source:MGI Symbol;Acc:MGI:1918180]	9871	-0.02721652388572693	0.18982303	No
Prkab1	protein kinase, AMP-activated, beta 1 non-catalytic subunit [Source:MGI Symbol;Acc:MGI:1336167]	10120	-0.03001508116722107	0.17797934	No
Sec61a1	SEC61 translocon subunit alpha 1 [Source:MGI Symbol;Acc:MGI:1858417]	10140	-0.030237730592489243	0.17800964	No
Rxra	retinoid X receptor alpha [Source:MGI Symbol;Acc:MGI:98214]	10374	-0.03317900747060776	0.16704942	No
F2r	coagulation factor II thrombin receptor [Source:MGI Symbol;Acc:MGI:101802]	10518	-0.03472026437520981	0.16080463	No
Epha2	Eph receptor A2 [Source:MGI Symbol;Acc:MGI:95278]	10520	-0.03474265709519386	0.16191886	No
Fgf13	fibroblast growth factor 13 [Source:MGI Symbol;Acc:MGI:109178]	10575	-0.035362742841243744	0.1603075	No
Ldhb	lactate dehydrogenase B [Source:MGI Symbol;Acc:MGI:96763]	10588	-0.03547583520412445	0.16087633	No
Upp1	uridine phosphorylase 1 [Source:MGI Symbol;Acc:MGI:1097668]	10797	-0.03798644617199898	0.15137294	No
Fdxr	ferredoxin reductase [Source:MGI Symbol;Acc:MGI:104724]	10989	-0.040372949093580246	0.14283055	No
Trp53	transformation related protein 53 [Source:MGI Symbol;Acc:MGI:98834]	11026	-0.04079430177807808	0.14233424	No
Ephx1	epoxide hydrolase 1, microsomal [Source:MGI Symbol;Acc:MGI:95405]	11205	-0.04283653572201729	0.13454819	No
Tspyl2	TSPY-like 2 [Source:MGI Symbol;Acc:MGI:106244]	11340	-0.04447093605995178	0.12909701	No
Iscu	iron-sulfur cluster assembly enzyme [Source:MGI Symbol;Acc:MGI:1913633]	11363	-0.04476983845233917	0.12945959	No
Polh	polymerase (DNA directed), eta (RAD 30 related) [Source:MGI Symbol;Acc:MGI:1891457]	11682	-0.048750266432762146	0.114617385	No
Krt17	keratin 17 [Source:MGI Symbol;Acc:MGI:96691]	11707	-0.0491231270134449	0.11502243	No
Rhbdf2	rhomboid 5 homolog 2 [Source:MGI Symbol;Acc:MGI:2442473]	11812	-0.050369344651699066	0.11132379	No
Ccnk	cyclin K [Source:MGI Symbol;Acc:MGI:1276106]	11876	-0.05114799365401268	0.109775856	No
Xpc	xeroderma pigmentosum, complementation group C [Source:MGI Symbol;Acc:MGI:103557]	12089	-0.05407830700278282	0.10060527	No
Lif	leukemia inhibitory factor [Source:MGI Symbol;Acc:MGI:96787]	12195	-0.05522289499640465	0.09701771	No
Hdac3	histone deacetylase 3 [Source:MGI Symbol;Acc:MGI:1343091]	12395	-0.0576968640089035	0.08864222	No
Cgrrf1	cell growth regulator with ring finger domain 1 [Source:MGI Symbol;Acc:MGI:1916368]	12524	-0.05934189632534981	0.084001064	No
Def6	differentially expressed in FDCP 6 [Source:MGI Symbol;Acc:MGI:1346328]	12711	-0.06151222065091133	0.076427266	No
Tprkb	Tp53rk binding protein [Source:MGI Symbol;Acc:MGI:1917036]	13100	-0.06672891229391098	0.058561146	No
Bak1	BCL2-antagonist/killer 1 [Source:MGI Symbol;Acc:MGI:1097161]	13160	-0.06761413812637329	0.057773136	No
Plk2	polo like kinase 2 [Source:MGI Symbol;Acc:MGI:1099790]	13176	-0.06783616542816162	0.059272606	No

Steap3	STEAP family member 3 [Source:MGI Symbol;Acc:MGI:1915678]	13469	-0.07183774560689926	0.04655256	No
Ccnd2	cyclin D2 [Source:MGI Symbol;Acc:MGI:88314]	13490	-0.07210949808359146	0.04793636	No
Sphk1	sphingosine kinase 1 [Source:MGI Symbol;Acc:MGI:1316649]	13531	-0.07284949719905853	0.048308622	No
Trp63	transformation related protein 63 [Source:MGI Symbol;Acc:MGI:1330810]	14110	-0.08152075111865997	0.021093372	No
Inhbb	inhibin beta-B [Source:MGI Symbol;Acc:MGI:96571]	14199	-0.08284419775009155	0.019313775	No
S100a10	S100 calcium binding protein A10 (calpactin) [Source:MGI Symbol;Acc:MGI:1339468]	14341	-0.08480686694383621	0.014853651	No
Tnni1	troponin I, skeletal, slow 1 [Source:MGI Symbol;Acc:MGI:105073]	14389	-0.08560756593942642	0.015291371	No
Mapkapk3	mitogen-activated protein kinase-activated protein kinase 3 [Source:MGI Symbol;Acc:MGI:2143163]	14442	-0.08658214658498764	0.015502707	No
Ralgds	ral guanine nucleotide dissociation stimulator [Source:MGI Symbol;Acc:MGI:107485]	14942	-0.09515644609928131	-0.0071612042	No
Sdc1	syndecan 1 [Source:MGI Symbol;Acc:MGI:1349162]	14944	-0.09519179165363312	-0.0040181456	No
Pdgfa	platelet derived growth factor, alpha [Source:MGI Symbol;Acc:MGI:97527]	15100	-0.09787815809249878	-0.008765028	No
Wrap73	WD repeat containing, antisense to Trp73 [Source:MGI Symbol;Acc:MGI:1891749]	15147	-0.09865996986627579	-0.007837417	No
Bax	BCL2-associated X protein [Source:MGI Symbol;Acc:MGI:99702]	15366	-0.10261981934309006	-0.015689744	No
Hras	Harvey rat sarcoma virus oncogene [Source:MGI Symbol;Acc:MGI:96224]	15480	-0.104886494576931	-0.018025016	No
Rrp8	ribosomal RNA processing 8 [Source:MGI Symbol;Acc:MGI:1914251]	15549	-0.10625553876161575	-0.017982494	No
Aen	apoptosis enhancing nuclease [Source:MGI Symbol;Acc:MGI:1915298]	15635	-0.10808397084474564	-0.018759524	No
Rad9a	RAD9 checkpoint clamp component A [Source:MGI Symbol;Acc:MGI:1328356]	15882	-0.11283370852470398	-0.027719975	No
Sesn1	sestrin 1 [Source:MGI Symbol;Acc:MGI:2155278]	15993	-0.11539991199970245	-0.029546935	No
Klf4	Kruppel-like transcription factor 4 (gut) [Source:MGI Symbol;Acc:MGI:1342287]	16138	-0.1191505715250969	-0.033009853	No
Vdr	vitamin D (1,25-dihydroxyvitamin D3) receptor [Source:MGI Symbol;Acc:MGI:103076]	16247	-0.12177347391843796	-0.034519263	No
Ccp110	centriolar coiled coil protein 110 [Source:MGI Symbol;Acc:MGI:2141942]	16291	-0.12282154709100723	-0.032625273	No
Tpd52l1	tumor protein D52-like 1 [Source:MGI Symbol;Acc:MGI:1298386]	16475	-0.1267264485359192	-0.03785486	No
Hmox1	heme oxygenase 1 [Source:MGI Symbol;Acc:MGI:96163]	16487	-0.1270924210548401	-0.034159325	No
Zfp365	zinc finger protein 365 [Source:MGI Symbol;Acc:MGI:2143676]	16824	-0.1356586515903473	-0.047017407	No
Cdh13	cadherin 13 [Source:MGI Symbol;Acc:MGI:99551]	17037	-0.14118650555610657	-0.053264424	No
Ninj1	ninjurin 1 [Source:MGI Symbol;Acc:MGI:1196617]	17288	-0.14806342124938965	-0.061249755	No
Vamp8	vesicle-associated membrane protein 8 [Source:MGI Symbol;Acc:MGI:1336882]	17296	-0.14839184284210205	-0.05663208	No
Clca2	chloride channel accessory 2 [Source:MGI Symbol;Acc:MGI:2139758]	17344	-0.14978274703025818	-0.05404048	No
Tm4sf1	transmembrane 4 superfamily member 1 [Source:MGI Symbol;Acc:MGI:104678]	17410	-0.15198247134685516	-0.05230779	No
Dnttip2	deoxynucleotidyltransferase, terminal, interacting protein 2 [Source:MGI Symbol;Acc:MGI:1923173]	17535	-0.15625141561031342	-0.05348913	No
Rgs16	regulator of G-protein signaling 16 [Source:MGI Symbol;Acc:MGI:108407]	17765	-0.16531400382518768	-0.05980729	No
Tsc22d1	TSC22 domain family, member 1 [Source:MGI Symbol;Acc:MGI:109127]	18194	-0.1861618459224701	-0.07573769	No
Rpl18	ribosomal protein L18 [Source:MGI Symbol;Acc:MGI:98003]	18356	-0.19594402611255646	-0.07750415	No
Hspa4l	heat shock protein 4 like [Source:MGI Symbol;Acc:MGI:107422]	18360	-0.19601671397686005	-0.07108079	No
Sfn	stratifin [Source:MGI Symbol;Acc:MGI:1891831]	18390	-0.19779826700687408	-0.065944925	No
Atf3	activating transcription factor 3 [Source:MGI Symbol;Acc:MGI:109384]	18785	-0.23192153871059418	-0.078577675	No
Ier3	immediate early response 3 [Source:MGI Symbol;Acc:MGI:104814]	18791	-0.23239921033382416	-0.07103687	No
Rnf19b	ring finger protein 19B [Source:MGI Symbol;Acc:MGI:1922484]	18828	-0.23807866871356964	-0.06491182	No
Cyfip2	cytoplasmic FMR1 interacting protein 2 [Source:MGI Symbol;Acc:MGI:1924134]	18945	-0.255736380815506	-0.06233965	No
Cebpa	CCAAT/enhancer binding protein alpha [Source:MGI Symbol;Acc:MGI:99480]	19090	-0.2809803783893585	-0.06037115	No
Serpinb5	serine (or cysteine) peptidase inhibitor, clade B, member 5 [Source:MGI Symbol;Acc:MGI:109579]	19092	-0.28132662177085876	-0.050980937	No
Sertad3	SERTA domain containing 3 [Source:MGI Symbol;Acc:MGI:2180697]	19123	-0.28737837076187134	-0.04289036	No
Gls2	glutaminase 2 (liver, mitochondrial) [Source:MGI Symbol;Acc:MGI:2143539]	19132	-0.28956225514411926	-0.03358647	No
H1f2	H1.2 linker histone, cluster member [Source:MGI Symbol;Acc:MGI:1931526]	19231	-0.3336242139339447	-0.027467443	No
Hbegf	heparin-binding EGF-like growth factor [Source:MGI Symbol;Acc:MGI:96070]	19370	-0.4776257872581482	-0.018588115	No
H2ac25	H2A clustered histone 25 [Source:MGI Symbol;Acc:MGI:2448458]	19436	-0.7437429428100586	0.0030055537	No
