# Supplementary material for: Targeting oncogene-induced senescence in ETV6::RUNX1 pre-leukemic cells
Source: Cell Death Discov. 2026 Mar 11;12:145. doi: 10.1038/s41420-026-03001-5 (PMC13039127; doi:10.1038/s41420-026-03001-5)
Supplement: Supplementary file 4 — Supplementary information [file 41420_2026_3001_MOESM4_ESM.docx]

**Supplementary Figures**


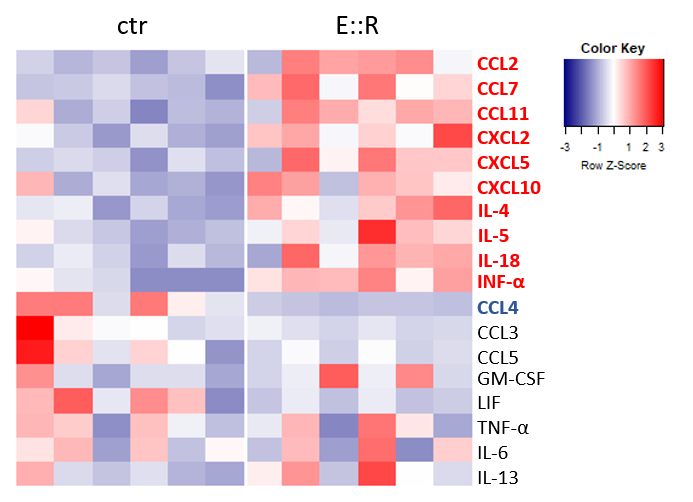


**Supplementary Figure S1**. **Heatmap showing all cytokines/chemokines detected in a multiplex assay performed using conditioned media collected from E::R and control cells.** Data are represented as Z-Score of normalized concentrations. Cytokines/Chemokines that are significantly differentially secreted (FDR<0.05) are highlighted in bold. Red and blue indicate up-regulated and down-regulated factors, respectively.


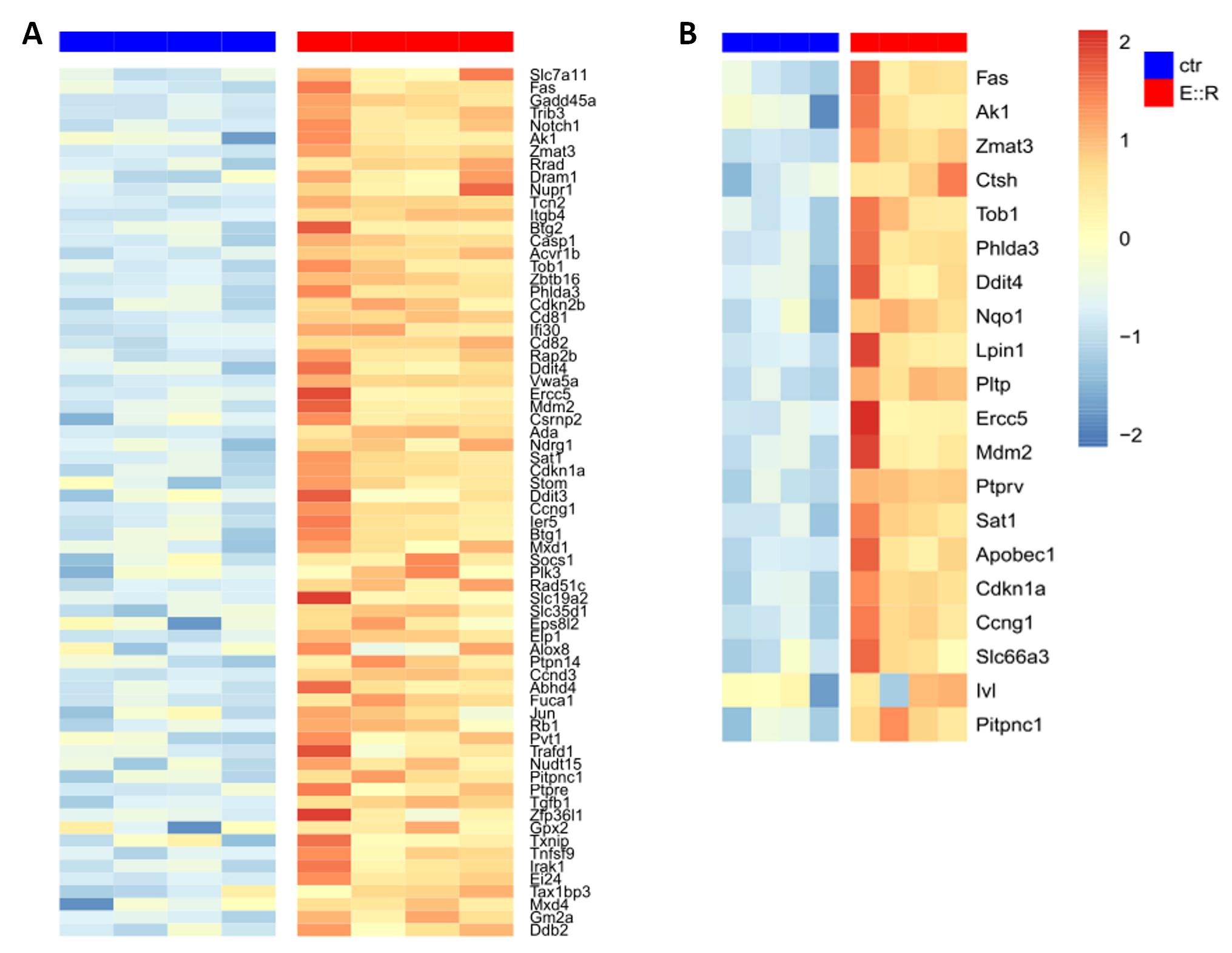


**Supplementary Figure S2**. **Heat maps of the gene set HALLMARK_P53_PATHWAY (A) ONGUSAHA_TP53_TARGETS (B) containing all genes found significantly up-regulated in E::R+ BaF3 cells vs control cells.**

**
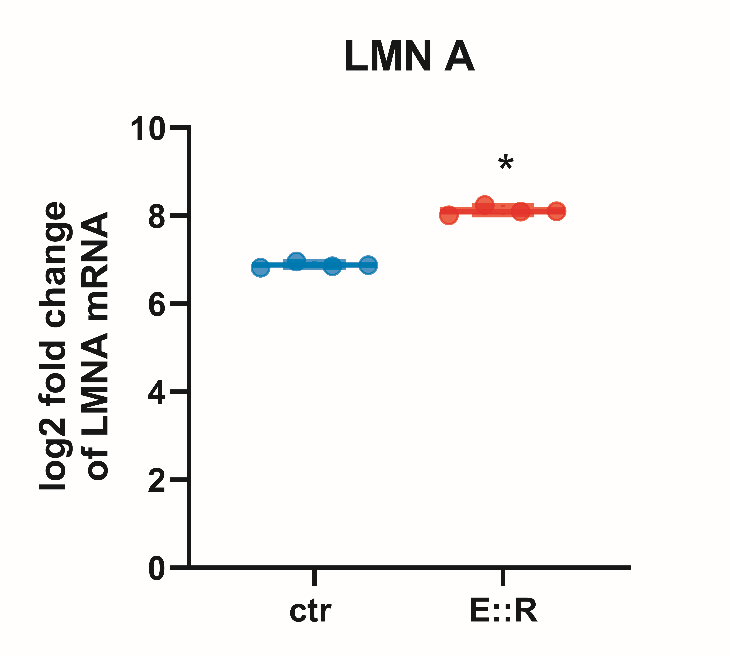
**

**Supplementary Figure S3. GEP analysis of *LMNA* gene in E::R+ and control cells.** Boxplot of LMNA gene expression level in E::R+ and control cells. Error bar represents SD. (FDR: < 0.05).
