# Supplementary material for: Targeting oncogene-induced senescence in ETV6::RUNX1 pre-leukemic cells
Source: Cell Death Discov. 2026 Mar 11;12:145. doi: 10.1038/s41420-026-03001-5 (PMC13039127; doi:10.1038/s41420-026-03001-5)

Original image: Figure 3A

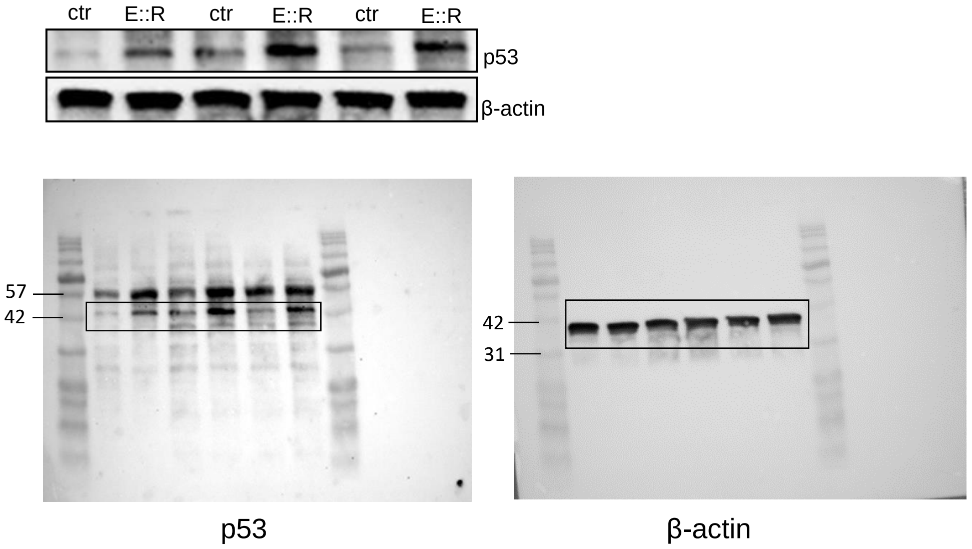

Original image: Figure 3B

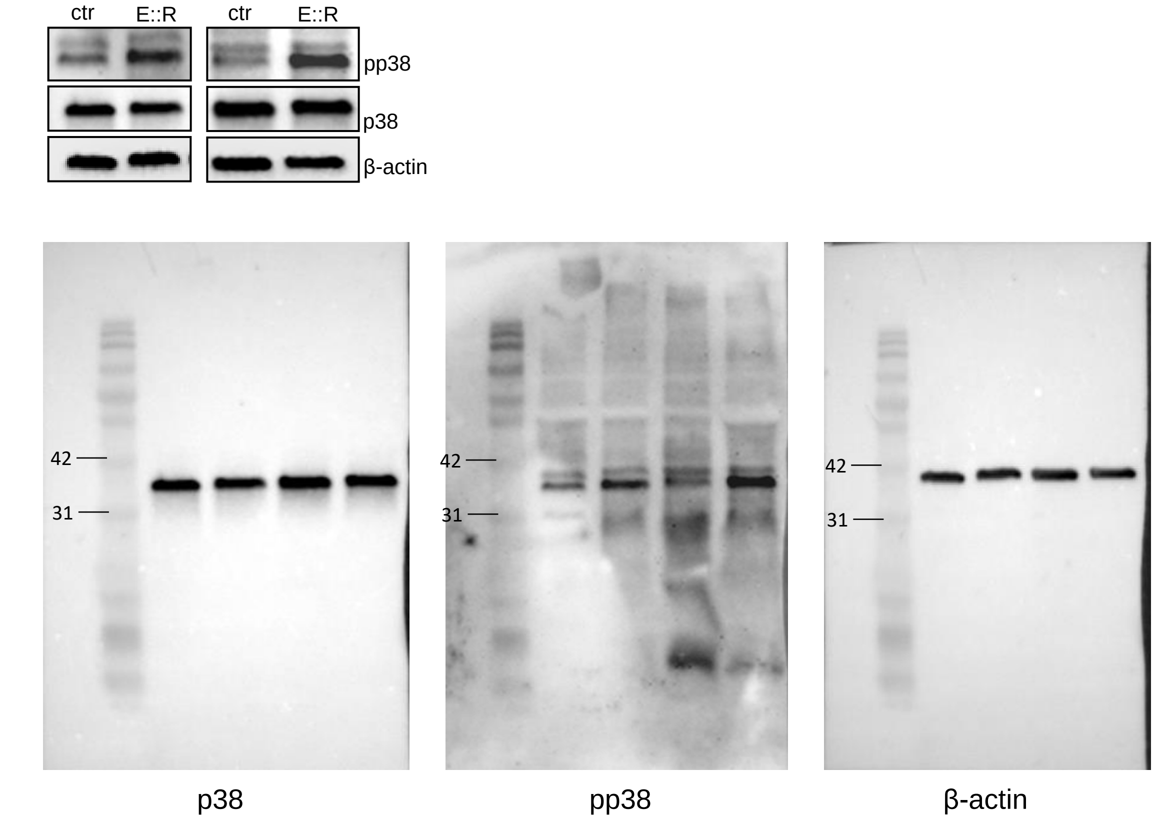

Original image: Figure 3C

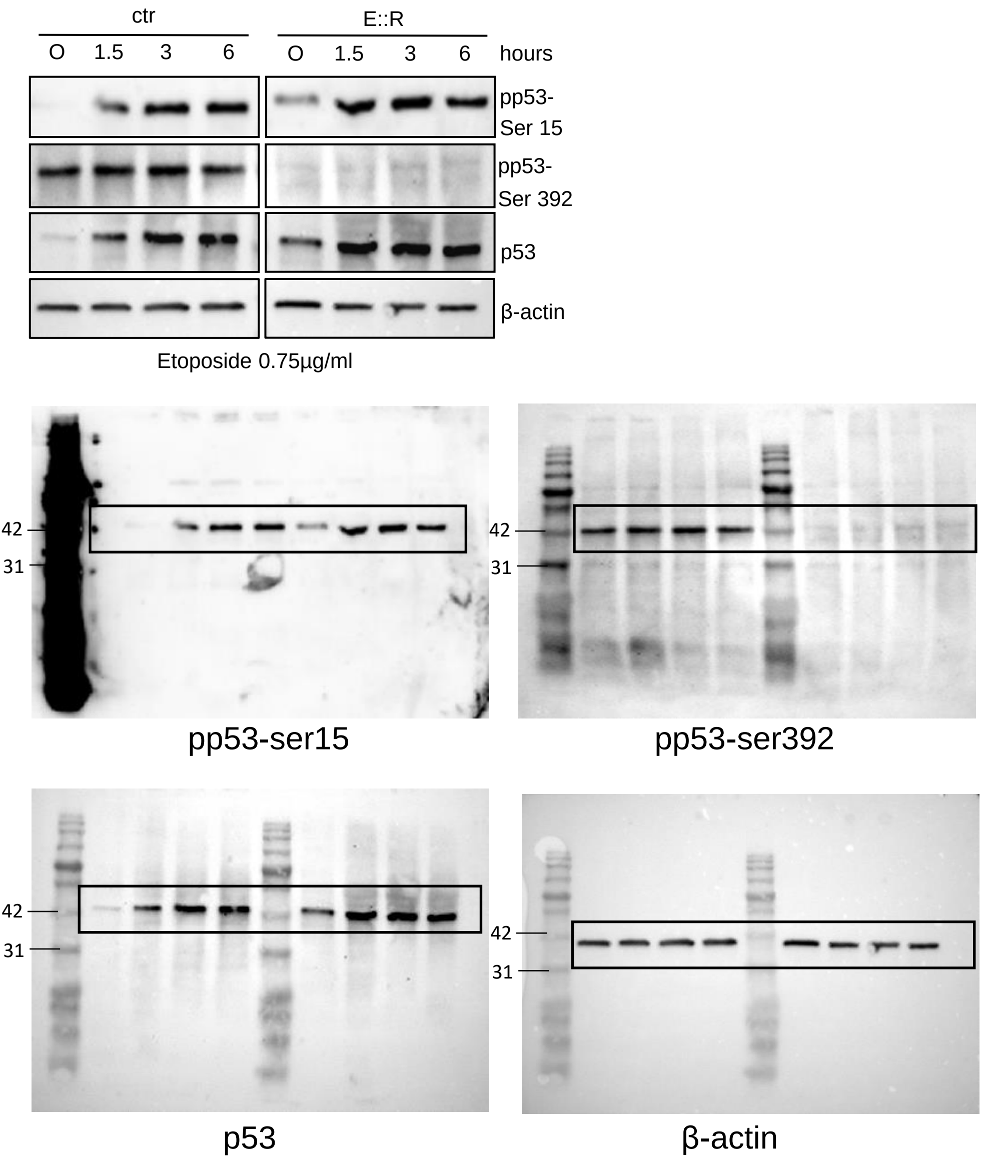

Original image: Figure 3D

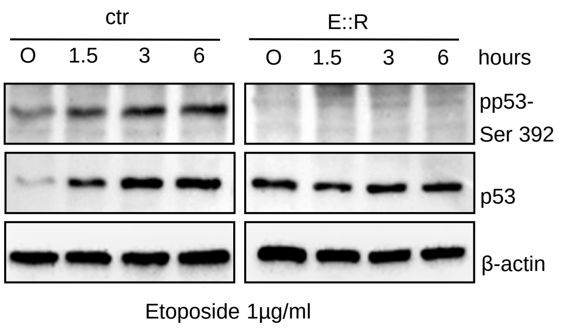

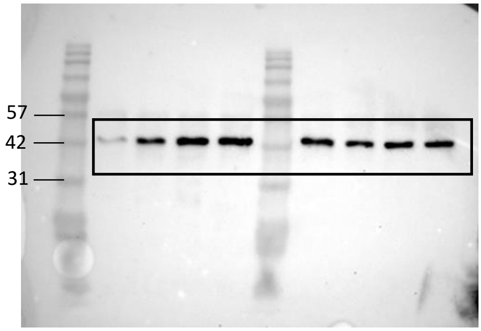

p53

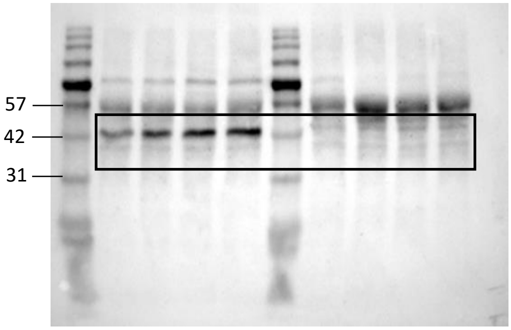

pp53-ser392

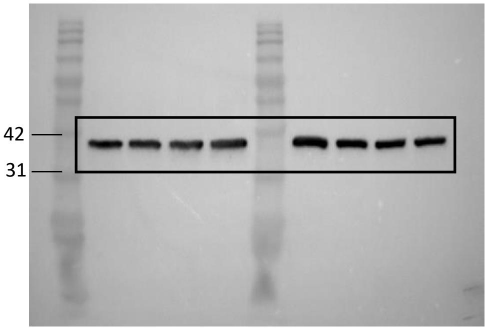

β-actin

Original image: Figure 3E

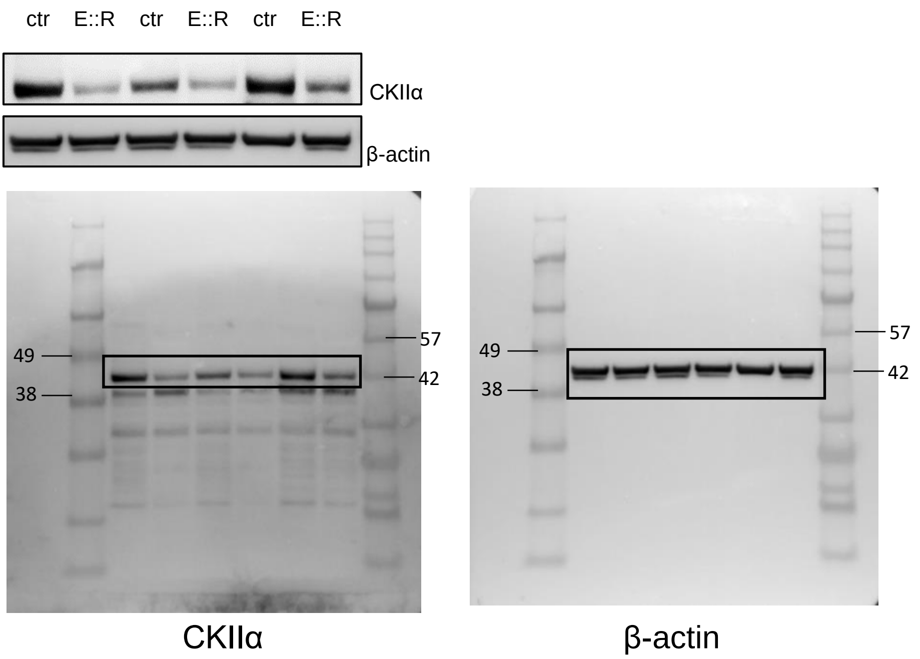

Original image: Figure 3F

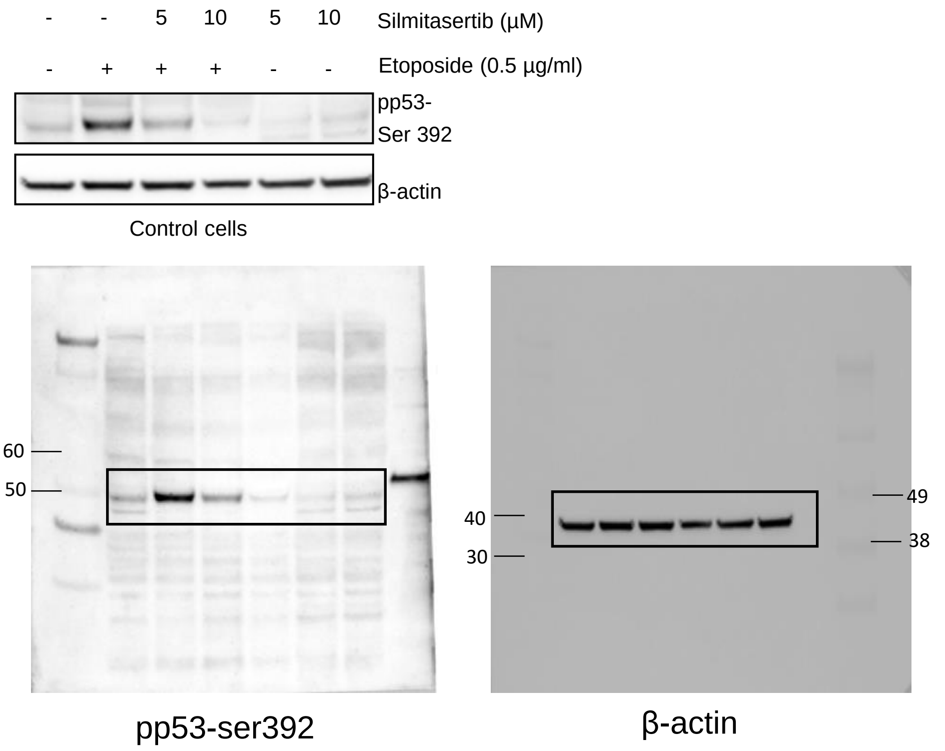

Supplement: Supplementary file 5 — Uncropped western blot images [file 41420_2026_3001_MOESM5_ESM.pdf]
